# Genome‐wide association analysis of serum alanine and aspartate aminotransferase, and the modifying effects of BMI in 388k European individuals

**DOI:** 10.1002/gepi.22392

**Published:** 2021-06-29

**Authors:** Chuan Gao, Anthony Marcketta, Joshua D. Backman, Colm O'Dushlaine, Jeffrey Staples, Manuel Allen Revez Ferreira, Luca A. Lotta, John D. Overton, Jeffrey G. Reid, Tooraj Mirshahi, Regeneron Genetics Center, Geisinger Regeneron Discovehr Collaboration, Aris Baras, Gonçalo Abecasis, Alan R. Shuldiner, Cristopher V. Van Hout, Shane McCarthy

**Affiliations:** ^1^ Regeneron Genetics Center Regeneron Pharmaceuticals Tarrytown New York USA; ^2^ Molecular and Functional Genomics Geisinger Clinic Danville Pennsylvania USA

**Keywords:** ALT, AST, GWAS, interaction, liver disease

## Abstract

Serum alanine aminotransferase (ALT) and aspartate aminotransferase (AST) are biomarkers for liver health. Here we report the largest genome‐wide association analysis to date of serum ALT and AST levels in over 388k people of European ancestry from UK biobank and DiscovEHR. Eleven million imputed markers with a minor allele frequency (MAF) ≥ 0.5% were analyzed. Overall, 300 ALT and 336 AST independent genome‐wide significant associations were identified. Among them, 81 ALT and 61 AST associations are reported for the first time. Genome‐wide interaction study identified 9 ALT and 12 AST independent associations significantly modified by body mass index (BMI), including several previously reported potential liver disease therapeutic targets, for example, *PNPLA3*, *HSD17B13*, and *MARC1*. While further work is necessary to understand the effect of ALT and AST‐associated variants on liver disease, the weighted burden of significant BMI‐modified signals is significantly associated with liver disease outcomes. In summary, this study identifies genetic associations which offer an important step forward in understanding the genetic architecture of serum ALT and AST levels. Significant interactions between BMI and genetic loci not only highlight the important role of adiposity in liver damage but also shed light on the genetic etiology of liver disease in obese individuals.

## 1 INTRODUCTION

Nonalcoholic fatty liver disease (NAFLD) is an epidemic in the United States with a prevalence between 30% and 40% among adults (Sharma & John, 2019; Spengler & Loomba, 2015). Although often benign, NAFLD may also progress to nonalcoholic steatohepatitis (NASH), which can lead to cirrhosis, liver failure, and liver cancer if left untreated (Adams et al., 2005). Obesity is a strong risk factor for NAFLD. The prevalence of NAFLD in normal‐weight (body mass index [BMI] < 25 kg/m^2^) men and women is on average 7.5% and 6.7%, respectively, compared with 57% and 44% in men and women with a BMI >35 kg/m^2^ (Yki‐Jarvinen, 2014). Although the pathophysiology between obesity and NAFLD is not fully understood, it has been hypothesized that fat accumulation in the liver may be linked to the exposure to free fatty acids and adipokines released from adipose tissue (Jakobsen et al., 2007).

Serum alanine aminotransferase (ALT) and aspartate aminotransferase (AST) are commonly measured biomarkers of liver health. Elevated ALT and AST levels are signatures of liver disease or damage, such as NAFLD, viral hepatitis, and drug‐induced liver damage (Kaplan, 2002). Serum ALT and AST levels are considered highly heritable with genetic factors explaining 20%–60% of the phenotypic variance (Makkonen et al., 2009; Rahmioglu et al., 2009; Sookoian & Pirola, 2015). Previous genome‐wide association studies (GWAS) identified numerous significant genetic loci associated with ALT and AST levels (Moon et al., 2019; Prins et al., 2017; Sinnott‐Armstrong et al., 2019; Young et al., 2019). In addition, some ALT and AST signals were reported to have obesity‐dependent effects. For example, *PNPLA3* and *HSD17B13* associations have been shown to have stronger effects in obese individuals (Abul‐Husn et al., 2018; Giudice et al., 2011; Mann & Anstee, 2017; Stojkovic et al., 2014). However, no genome‐wide agnostic screening of obesity‐dependent effects has been performed.

Here we report a GWAS of serum ALT and AST levels in 388k unrelated individuals of European ancestry from UKB and DiscovEHR. We also report the first genome‐wide interaction study (GWIS) to investigate the effect of BMI on ALT and AST genetic associations. Finally, we show that ALT‐ and AST‐associated variants that are significantly modified by BMI may have an important impact on the risk of liver disease risks, for example, fatty liver disease, shedding light on the development of potential therapeutics.

## 2 METHODS

### 2.1 UK Biobank (UKB) data

A detailed description of the UKB study design, and collection of phenotypic and genotype data has been published previously by UKB (Bycroft et al., 2018). Consenting individuals participating in the UKB study were genotyped using the Affymetrix UK Biobank Axiom Array and the UK BiLEVE Axiom Array. Genotype imputation was performed centrally by UKB based on a merged reference panel incorporating UK 10 K, 1000 Genome, and Haplotype Reference Consortium (HRC). Imputed variants were then filtered based on minor allele frequency (MAF ≥ 0.5%) and Hardy–Weinberg (*p* < 10 × 10^−15^). Individuals of European ancestry were identified using a linear model trained based on PC estimates from HapMap3. Overall, 319,882 unrelated individuals of European ancestry were included for analysis of two enzyme levels: ALT and AST. Serum levels of ALT and AST from the initial visit (2006–2010) were measured centrally by UKB based on International Federation of Clinical Chemistry (IFCC). A description of the UKB sample demographics is shown in Table S1. Further information about the UKB sample collection and each phenotype can also be found via the UKB Showcase website (https://biobank.ndph.ox.ac.uk/showcase/).

### 2.2 DiscovEHR data

A detailed description of the DiscoverEHR study design has been published previously (Dewey et al., 2016). In short, the DiscovEHR cohort is a subset of individuals enrolled in the Geisinger Healthcare system who consented to participate in Geisinger's MyCode Community Health Initiative. Genomic DNA samples were transferred to the Regeneron Genetics Center from the Geisinger Health System. Genotyping was performed at the Regeneron Genetics Center in two waves. In the first wave, individuals were genotyped using the Illumina Human OmniExpressExome array (8v1‐2). In the second wave, genotyping was performed using the llumina Global Screening Array. These two waves are referred as “DiscovEHR OMNI” and “DiscovEHR GSA,” respectively. All analyses were performed in each cohort separately.

Individuals of European ancestry were identified using a linear model trained based on PC estimates from HapMap3. Pairwise identity‐by‐decent (IBD) estimates were calculated using PLINK2 (Purcell et al., 2007) and pedigrees were reconstructed using PRIMUS (Staples et al., 2014) as described previously (Dewey et al., 2016). Genotype imputation of European individuals was performed separately for DiscovEHR OMNI and GSA using the Michigan Imputation Server (Das et al., 2016) based on the HRC hg19 reference panel. Imputed variants were mapped (lifted over) to GRCh38/hg38, and then filtered based on MAF (MAF ≥ 0.5%), Hardy‐Weinberg (*p* < 10 × 10^−15^), and imputation info score (≥0.3). A total of 30,980 and 38,003 unrelated European individuals with DiscovEHR OMNI DiscovEHR GSA data, respectively, were included for analysis of serum ALT and AST levels. The median of serially measured laboratory values was selected for analysis following removal of likely spurious values that were >3 standard deviations from the intra‐individual median value. Age was defined as age at last encounter.

### 2.3 Statistical analysis

Genome‐wide associations analysis (GWAS) of ALT and AST were tested within each cohort using linear regression in PLINK2 (Purcell et al., 2007). Rank inverse normalized ALT and AST residuals were used for analyses after regressing out Age, Age^2^, Sex, the first 10 principle components, UKB‐specific covariates (study site and array, only adjusted in UKB), and BMI (to minimize the discovery of ALT and AST associations confounded by BMI). The rank inverse normalized residuals (RINT) were then tested for association based on the model

$$Y\sim \beta_{0}+\beta_{1}G,$$

where *Y* is the RINT residuals of ALT or AST, and *G* is the dosage genotype.

Genome‐wide interaction analysis (GWIS) was performed using linear regression in PLINK2 (24). Rank inverse normalized ALT and AST residuals were used for analyses after regressing out Age, Age^2^, Sex, the first 10 principle components, and UKB‐specific covariates. BMI was not used for residualization but was instead included as the interaction variable (INT) in the interaction model:

$$Y\sim \beta_{0}+\beta_{1}G+\beta_{2}INT+\beta_{3}G\times INT,$$

where, *Y* is the RINT ALT and AST residuals, *G* is the dosage genotype.

Summary statistics for the UKB and DiscovEHR cohorts were jointly meta‐analyzed after genomic correction using the fixed effect inverse variance weighted method implemented in METAL (Willer et al., 2010). Specifically, GWAS genomic correction was performed based on the LDSC regression intercept within each cohort (Bulik‐Sullivan et al., 2015); in GWIS, since LDSC intercept has not been tested as a genomic correction factor in interaction models, genomic correction was performed based on inflation factor (lambda). After meta‐analysis, no major inflation was detected (Table S2) and therefore post meta‐analysis genomic correction was not performed. HLA region was removed in Manhattan plots but were included for analyses.

### 2.4 Genome‐wide significant variants and signals

GCTA COJO was performed on meta‐analyzed GWAS and GWIS data, respectively, to identify a set of independently associated signals in each data set (31). A 10 Mb window was selected around signals with *p* values less than 5 × 10^−8^. The default settings for collinearity (*R* ^2^ > 0.9) and allele frequency differences (>0.2) were selected. Linkage disequilibrium (LD) estimates were derived from a random selection of 10 K unrelated European individuals in UKB. A locus is defined as a 1 Mb region. A novel signal is defined with a *r* ^2^ < 0.1 and at least 1 Mb away from any previously reported ALT or AST GWAS hits (ALT and AST GWAS catalog (Buniello et al., 2019) and a recent UKB study published on bioarchive (Sinnott‐Armstrong et al., 2019). A significant GTEx expression quantitative trait locus (eQTL) is defined based on the GTEx Portal accessed on 12/09/2020 (dbGaP accession number phs000424. vN. pN) with a *p* < 9.80 × 10^−10^ (Bonferroni correction of the genome‐wide significance threshold based on 51 tissue types) in at least one of the issue types (GTEx Consortium, 2015).

### 2.5 Gene–gene interaction analysis

Interaction between *PNPLA3* p.I148M and all GCTA COJO selected independent ALT and AST signals were tested. Similar to GWIS, a linear regression model was performed in PLINK2 (24). Rank inverse normalized ALT and AST residuals were analyzed after Age, Age^2^, Sex, BMI, the first 10 principle components, and UKB‐specific covariates were regressed out. The *PNPLA3* p.I148M genotype was coded as 0, 1, 2 and was included as the interaction variable in the model below:

$$Y\sim \beta_{0}+\beta_{1}G+\beta_{2}INT+\beta_{3}G\times INT,$$

where, *Y* is the RINT ALT and AST residuals, and G is the dosage genotype. A significant interaction signal is defined using a Bonferroni corrected *p* value threshold.

### 2.6 Polygenic risk score (PRS)

Independent association signals identified by GCTA COJO were used to construct PRS according to the formula:

$$PRS_{i}=\overset{M}{\underset{j}{\sum }}\beta_{j}\times Allele_{ij}.$$

The PRS for a given individual *i* is the sum product of the associated effect size (*β*) times the number of alternative (effect) alleles at all sites *j*. Scores were then transformed to a normal distribution with *N* (0,1). PRS associations are reported in standard deviation units.

### 2.7 Expression enrichment analysis

Independent association variants were mapped to genes if: (1) had a coding COJO variant, (2) had a coding variant in LD with a COJO variant or, (3) had an eQTL in LD with a COJO variant (but not in LD with a coding variant). Tissue expression enrichment analysis was performed using FUMA (Watanabe et al., 2017). In brief, 30 general tissue type tissue‐specific expression patterns were derived from GTEx v8 RNA‐seq data (GTEx Consortium, 2015). Upregulated gene‐set enrichment was tested and Benjamini–Hochberg (FDR) was used to control for multiple testing. Only gene sets which overlap with ≥2 genes with the input list are reported.

### 2.8 Liver disease associations

A total of six liver disease traits were selected for associations: fatty liver (K760), Cirrhosis, Fibrosis or Cirrhosis, NALD Cirrhosis, NALD Composite, NASH‐NAFLD Composite. The definition and number of cases for each liver disease trait in UKB is summarized in Table S12. Mixed effect associations were computed with the same set of imputed markers using SAIGE (Zhou et al., 2018). Since SAIGE accounts for relatedness, the entire European data set instead of the unrelated subset was analyzed. Age, Age^2^, Sex, Age × Sex, first 10 principle components, and UKB‐specific covariates were adjusted. A fixed effect inverse variance weighted meta‐analysis was performed using metal.

## 3 RESULTS

### 3.1 UKB and DiscovEHR

In total, 11 million imputed variants from 388,865 unrelated European individuals were analyzed for associations with ALT and AST levels. Sample demographics are summarized in Table S1. In UKB, 319,882 unrelated European individuals (53.7% females) were analyzed with 23.8% of the individuals being obese (BMI > 30 kg/m^2^). In DiscovEHR, 68,983 unrelated European individuals were included from DiscovEHR OMNI (*N* = 30,980) and DiscovEHR GSA (*N* = 38,003), respectively. Compared to UKB, DiscovEHR cohorts have proportionally more females (57.9% in OMNI and 61.3% in GSA) and a higher prevalence (50.2%) of obesity (Table S1).

### 3.2 Genome‐wide association analysis of serum ALT and AST levels

**Figure 1:**
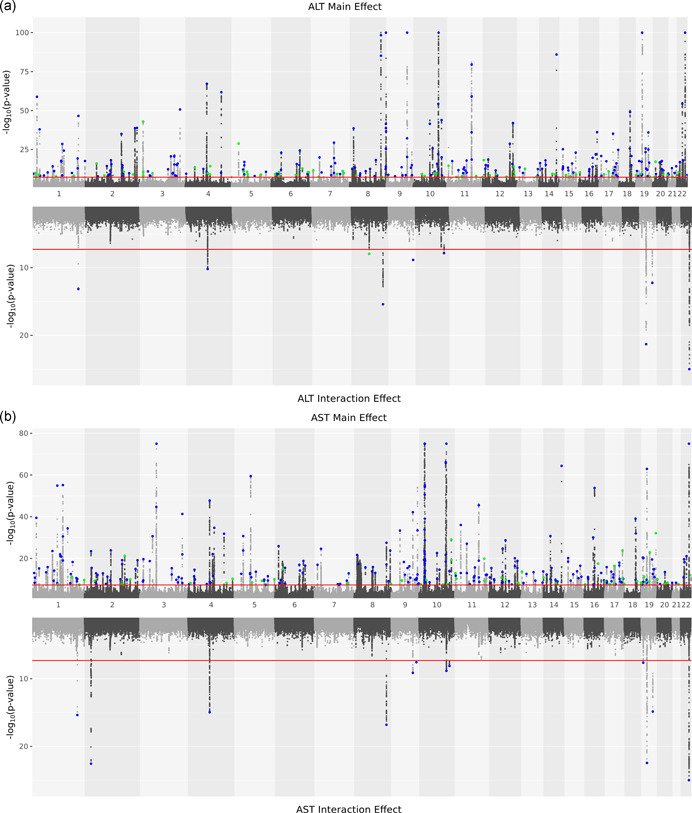
Manhattan plots of ALT and AST genome‐wide associations. (a) Manhattan plots of ALT genome‐wide associations. ALT GWAS main effects are plotted at the top; BMI interaction effects are plotted at the bottom. GCTA COJO selected variants are highlighted. Previously reported signals are highlighted in blue; novel signals are highlighted in green (defined as *R* ^2^ < 0.1 with any previously reported signals and at least 1 Mb away from any previously reported signals). For visualization, main effect *p* values are capped at 1E−100, interaction *p* values are capped at 1E−25. HLA region was excluded in the plot. (b) Manhattan plots of AST genome‐wide associations. AST GWAS main effects are plotted at the top; BMI interaction effects are plotted at the bottom. GCTA COJO selected variants are highlighted. Previously reported signals are highlighted in blue; novel signals are highlighted in green (defined as *R* ^2^ < 0.1 with any previously reported signals and at least 1 Mb away from any previously reported signals). For visualization, main effect *p* values are capped at 1E−75, interaction *p* values are capped at 1E−25. HLA region was excluded in the plot. ALT, alanine aminotransferase; AST, aspartate aminotransferase; GWAS, genome‐wide association studies

GWAS of ALT and AST was performed in DiscovEHR and UKB separately. In the meta‐analysis of the summary statistics from each study, 26,366 ALT and 43,727 AST variants reached genome‐wide significance (*p* < 5 × 10^−8^) (Figures 1 and S1 and Table S2). SNP‐heritability estimates for ALT and AST were approximately 19.09% (*SE*: 0.0131) and 21.75% (*SE*: 0.0215), respectively (Bulik‐Sullivan et al., 2015). Conditional analysis using GCTA COJO identified 300 ALT and 336 AST independent associations (from 255 to 268 loci) (Tables S3 and S4). Of these, 55 ALT and 71 AST variants are coding or in strong LD (*r* ^2^ > 0.8) with a coding variant based on Ensembl 85 gene model. Also, 172 ALT and 187 AST signals are in strong linkage disequilibrium (LD) with a significant GTEx expression quantitative trait locus (eQTL) (*p* < 9.80 × 10^−10^, after Bonferroni correction of the number of tissue types, Tables S3 and S4) (GTEx Consortium, 2015).

As expected, GWAS identified multiple previously reported liver enzyme associations. For example, rs738409 in patatin‐like phospholipase domain‐containing protein 3 (*PNPLA3*) gene (p.I148M, *p*_ALT_ = 4.15 × 10^−402^, *p*_AST_ = 1.03 × 10^−344^, Figure S2) is associated with 1.66 and 1.02 units higher ALT and AST levels (Romeo et al., 2008). Similarly, rs10433937 in 17 *β*‐hydroxysteroid dehydrogenase type 13 (*HSD17B13*) gene (*p*_ALT_ = 6.31 × 10^−68^) is significantly associated with lower ALT levels (Abul‐Husn et al., 2018). In addition, 81 ALT and 61 AST variants are reported for the first time (having a *r* ^2^ < 0.1 and at least 1 Mb away from any previously reported ALT or AST GWAS hits, see detail in method). The most significant novel association observed is an intronic variant within the gene peroxisome proliferator‐activated receptor gamma (*PPARG*, rs13083375, *p*_ALT_ = 1.04 × 10^−43^, Figure S3), lowering ALT by 0.523 units per allele in an additive genetic model. A complete list of novel signals is summarized in Tables S3 and S4.

### 3.3 GWIS of BMI‐dependent effects

A GWIS was performed to identify ALT‐ and AST‐associated loci with BMI‐dependent effects. In total, 571 ALT and 951 AST variants with significant BMI interactions were identified (*p *value for interaction (*p*_INT_) < 5 × 10^−8^, Figures 1 and S1 and Table S2). After conditional analysis, 9 ALT and 12 AST independent signals were observed (Tables 1 and 2). Among them, 4 ALT and 6 AST signals are either coding or in strong LD (*r* ^2^ > 0.8) with a coding variant; 5 ALT and 8 AST signals are in strong LD with a significant GTEx eQTL (*p* < 9.80 × 10^−10^, Tables S5 and S6).

**Table 1:** Meta‐analysis results of genome‐wide significant ALT BMI‐interaction effect association signals

				Main effect			BMI interaction		
MarkerName^a^	Gene	Annotation	AAF^b^	*β* (*SE*)	*p*	Direction^c^	*β* (*SE*)	*p*	Direction
1:220796686:A:G	*MARC1*	Missense	0.7017	0.0373 (0.0026)	2.52E−47	+++	0.0182 (0.0024)	7.08E−14	+++
4:87292732:T:C	*HSD17B13/*−	Intergenic	0.4324	−0.0415 (0.0024)	1.20E−67	−−−	−0.0147 (0.0022)	6.30E−11	−−−
8:58480714:A:G	*CYP7A1/*−	Intergenic	0.6605	−0.0044 (0.0025)	0.07531	‐++	−0.0134 (0.0023)	1.10E−08	−−−
8:125469835:A:G	*TRIB1/‐*	Intergenic	0.5061	−0.0509 (0.0024)	7.58E−103	−−−	−0.0181 (0.0022)	3.84E−16	−−−
9:129804387:G:A	*TOR1B*	Intronic	0.0951	−0.0306 (0.0041)	4.59E−14	−−−	−0.0231 (0.0038)	1.40E−09	−−−
10:112142660:A:C	*GPAM*	Intronic	0.2475	0.036 (0.0027)	2.57E−39	+++	0.0147 (0.0026)	1.39E−08	+++
19:19349732:G:C	*MAU2*	Intronic	0.0708	0.1005 (0.0046)	4.22E−105	+++	0.0422 (0.0044)	4.86E−22	+++
19:44908684:T:C	*APOE*	Missense	0.1519	−0.0417 (0.0033)	1.04E−36	−−−	−0.0223 (0.0031)	5.59E−13	−−−
22:43928850:C:T	*PNPLA3*	Missense	0.2191	0.1220 (0.0028)	4.15E−402	+++	0.0588 (0.0027)	8.32E−107	+++

Abbreviations: ALT, alanine aminotransferase; BMI, body mass index.

^a^ MarkerName is based on chromosome number, position (hg38), reference, and alternative/effect alleles.

^b^ Alternative/effect allele frequency.

^c^ Direction of the effect across UKB, DiscovEHR OMNI, and DiscovEHR GSA.

**Table 2:** Meta‐analysis results of genome‐wide significant AST BMI‐interaction effect association signals

				Main effect			BMI interaction		
MarkerName^a^	Gene	Annotation	AAF^b^	*β*​*​* (*SE*)	*p*	Direction^c^	*β*​*​* (*SE*)	*p*	Direction
1:220797157:A:G	*MARC1*	Intronic	0.6854	0.0152 (0.0026)	2.49E−09	+++	0.0206 (0.0025)	4.30E−16	+++
2:27508073:T:C	*GCKR*	Missense	0.6004	−0.0246 (0.0024)	3.53E−24	−−−	−0.0239 (0.0024)	2.78E−23	−−−
4:87292006:C:T	*HSD17B13/−*	Intergenic	0.4277	−0.0351 (0.0024)	2.92E−48	−−−	−0.0191 (0.0024)	1.11E−15	−−−
8:125469835:A:G	*TRIB1/*−	Intergenic	0.5061	−0.024 (0.0024)	5.20E−24	−−−	−0.0201 (0.0024)	1.57E−17	−−−
9:114383763:C:G	*AKNA*	Intronic	0.4853	−0.0324 (0.0024)	9.01E−42	−−−	−0.0146 (0.0024)	7.30E−10	−−−
9:129804387:G:A	*TOR1B*	Intronic	0.0951	−0.0261 (0.0041)	1.52E−10	−−−	−0.0225 (0.004)	2.74E−08	−−−
10:100174478:C:T	*ERLIN1*	Intronic	0.4379	−0.0458 (0.0024)	2.75E−81	−−−	−0.0144 (0.0024)	1.48E−09	−−−
10:112187282:C:T	*GPAM*	Upstream	0.7193	−0.0208 (0.0027)	5.73E−15	−−−	−0.0153 (0.0026)	7.70E−09	−−−
19:7222366:G:C	*INSR*	Intronic	0.5818	0.0123 (0.0024)	3.25E−07	+++	0.0134 (0.0024)	2.33E−08	+++
19:19349732:G:C	*MAU2*	Intronic	0.0708	0.0742 (0.0046)	1.38E−57	+++	0.0459 (0.0046)	3.57E−23	+++
19:44888997:C:T	*NECTIN2*	3′ UTR	0.1702	0.003 (0.0032)	0.3392	−++	−0.025 (0.0031)	1.39E−15	−−−
22:43928847:C:G	*PNPLA3*	Missense	0.2191	0.1136 (0.0029)	1.03E−344	+++	0.0697 (0.0028)	2.95E−133	+++

Abbreviations: AST, aspartate aminotransferase; BMI, body mass index.

^a^ MarkerName is based on chromosome number, position (hg38), reference, and alternative/effect alleles.

^b^ Alternative/effect allele frequency.

^c^ Direction of the effect across UKB, DiscovEHR OMNI, and DiscovEHR GSA.

**Figure 2:**
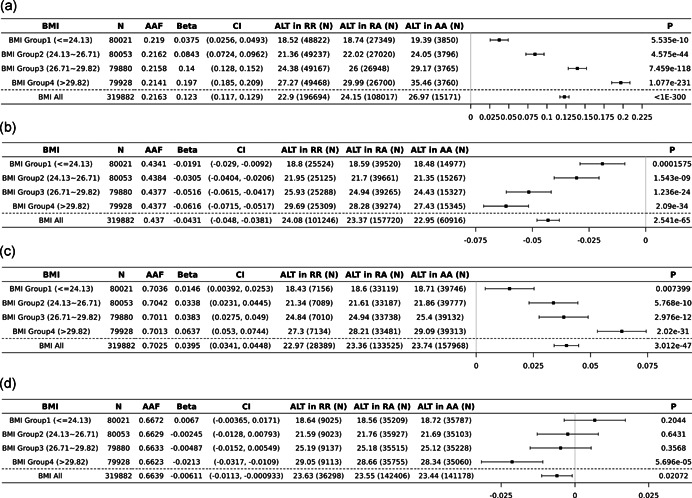
Forest plot of *PNPLA3*, *HSD17B13*, *MARC1*, and *CYP7A1* associations with ALT, stratified by BMI groups. (a) *PNPLA3* I148M (22:43928847:C:G) association with ALT, stratified by BMI groups. (b) *HSD17B13* (4:87292732:T:C) association with ALT, stratified by BMI groups. (c) *MARC1* (1:220796686:A:G) association with ALT, stratified by BMI groups. (d) *CYP7A1* (8:58480714:A:G) association with ALT, stratified by BMI groups. Association was analyzed with RINTed phenotypes in UKB with the adjustment of age, age2, sex, BMI, 10PCs, and study‐specific covariates. BMI groups are defined based on the 25% quartiles of BMI distribution. ALT, alanine aminotransferase; BMI, body mass index

GWIS identified several previously reported BMI‐modified signals, for example, *PNPLA3*, *HSD17B13* (Abul‐Husn et al., 2018; Giudice et al., 2011; Mann & Anstee, 2017; Stojkovic et al., 2014). The most significant BMI interaction was detected at rs738409 in *PNPLA3* (p.I148M, *p*_ALT_INT_ = 8.32 × 10^−107^, *p*_AST_INT_ = 2.95 × 10^−133^). In the highest BMI quartile (top 25%, BMI > 29.82 kg/m^2^), the effect of alternate allele (G) is 10‐fold greater (3.37 units/allele) than the effect observed in the low BMI quartile (bottom 25%, BMI < 24.13 kg/m^2^) (Figure 2). Similarly, rs6811902 in *HSD17B13* is also significantly modified by BMI (*p*_ALT_INT_ = 6.30 × 10^−11^, *p*_AST_INT_ = 1.11 × 10^−15^) where the alternate allele (C) is associated with a greater effect on lowering ALT and AST in individuals with elevated BMI relative to the low BMI quartile (Figure 2).

In addition, the GWIS also identified novel BMI‐dependent associations in previously reported liver disease loci. For example, consistent with previous reports (Emdin et al., 2020), the alternative allele (G) of the missense variant rs2642438 (p.T165A) in mitochondrial amidoxime reducing component 1 (*MARC1*) is associated with higher ALT and AST levels (*p*_ALT_ = 2.52 × 10^−47^, *p*_AST_ = 6.24 × 10^−11^). The associations were significantly modified by BMI (*p*_ALT_INT_ = 7.08 × 10^−14^, *p*_AST_INT_ = 4.70 × 10^−16^) and a greater effect was observed in the higher BMI quartile. On average, the alternative allele is associated with 0.128 units higher ALT in the low BMI quartile and 0.935 units higher ALT in the high BMI quartile (Figure 2). Similarly, significant BMI‐dependent effects were also observed in variants from gene MAU2 sister chromatid cohesion factor (*MAU2*) and tribbles pseudokinase 1 (*TRIB1*) (Tables S5 and S6).

GWIS also identified a novel BMI interaction with insignificant main effect association. An intergenic variant (rs4738684) near gene cytochrome P450 family 7 subfamily A member 1 (*CYP7A1*) was identified with a significant BMI interaction effect (*p*_INT_ = 1.10 × 10^−8^). The alternative allele (G) is associated with lower ALT level only in the high BMI quartile and no significant effect is detected in the low BMI individuals (Figures 2 and S5). *CYP7A1* encodes a protein that catalyzes the first reaction in the cholesterol catabolic pathway and converts cholesterol to bile acids, which is the primary mechanism for the removal of cholesterol from the body (O'Leary et al., 2016). However, it is still unclear why observed ALT association is only present in high BMI individuals and no effect is observed in low BMI individuals.

### 3.4 Gene × Gene interaction with *PNPLA3* I148M

**Figure 3:**
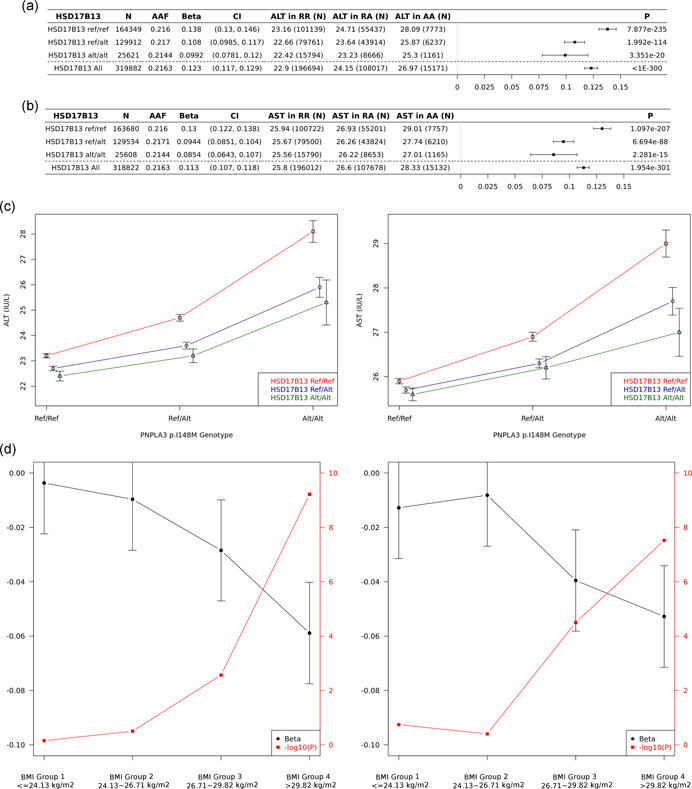
*PNPLA3 I148M* association with ALT and AST, stratified by *HSD17B13* genotype. (a) *PNPLA3 I148M* ALT associations, stratified by *HSD17B13* (*p*_INT_ = 3.19E−07). (b) *PNPLA3 I148M AST associations, stratified by HSD17B13* (*p*_INT_ = 4.91E−09). Association was analyzed with RINTed phenotypes in UKB with the adjustment of age, age2, sex, BMI, 10PCs, and study‐specific covariates. BMI groups are defined based on the 25% quartiles of BMI distribution. (c) *HSD17B13* protective alleles partially protect *PNPLA3* I148M risk. (d) *PNPLA3* Interaction with *HSD17B13*, stratified by BMI groups (left: ALT; right: AST). ALT, alanine aminotransferase; AST, aspartate aminotransferase; BMI, body mass index

Independently associated ALT (*N* = 300) and AST (*N* = 336) signals were evaluated for genetic interactions with *PNPLA3* p.I148M, as a proxy for their therapeutic potential in *PNPLA3* risk allele carriers. Only *HSD17B13* variants (rs10433937, *p*_ALT_INT_ = 3.19 × 10^−7^; rs13117201, *p*_AST_INT_ = 4.91 × 10^−9^) met the stringent Bonferroni corrected significant threshold (Tables S7 and S8). The magnitude of the per *PNPLA3* p.I148M allele increase in ALT and AST was significantly lowered by *HSD17B13* genotype. On average, per *HSD17B13* allele reduces the *PNPLA3* p.I148M allelic effect on ALT by 21%. Interestingly, a greater effect was observed in high BMI quartile (Figure 3).

### 3.5 Tissue expression enrichment analysis

ALT and AST association signals were mapped to genes to evaluate tissue expression enrichment. ALT‐associated genes are significantly enriched among several tissue types including liver (2.01 × 10^−17^) and adipose tissue (6.37 × 10^−14^). Adipose tissue is consistently upregulated in genes mapped from novel (6.64×10^‐5^) and previously reported (2.51 × 10^−14^) ALT associations (Figure S6 and Table S9). Similarly, genes mapped from AST‐associated variants are enriched in lung consistently between novel (2.43 × 10^−4^) and previously reported (7.64 × 10^−16^) signals. (Figure S7 and Table S9). Notably, genes with significant BMI‐dependent ALT‐ and AST‐associated variants are enriched in liver (*p*_ALT_ = 3.14 × 10^−5^, *p*_AST_ = 5.41 × 10^−6^) and adipose tissues (*p*_ALT_ = 1.42 × 10^−3^, *p*_AST_ = 3.02 × 10^−4^) only (Figures S6 and S7).

### 3.6 ALT, AST BMI‐interaction signals and liver disease

We also investigated the impact of ALT (*N* = 300) and AST (*N* = 336) associated variants on six liver disease traits. Specifically, this analysis focused on fatty liver, cirrhosis, fibrosis/cirrhosis, NALD Cirrhosis, NALD Composite, NASH‐NAFLD Composite. Twelve ALT and 13 AST signals had a *p* value less than 1 × 10^−5^ (rounded *p* value threshold based on Bonferroni correction) in at least one of the liver disease traits (Tables S3 and S4). As expected, the most significant liver disease association was identified at a previously reported missense variant (*PNPLA3*, rs738409) having increased risks with multiple liver disease conditions including NASH‐NAFLD composite (odds ratio [OR] = 1.713, *p* = 6.21 × 10^−136^) and fibrosis/cirrhosis (OR = 1.484, *p* = 2.99 × 10^−115^) (Tables S3 and S4). Among the novel liver enzyme associations, a missense variant (rs3816873, p.I128T, *p*_ALT_ = 4.15 × 10^−15^, *p*_AST_ = 3.16 × 10^−12^) in the gene microsomal triglyceride transfer protein (*MTTP*) has the most significant association with liver disease traits (NASH NAFLD composite, OR = 0.921, *p* = 3.33 × 10^−5^) (Figure S4 and Table S3).

As expected, not all liver enzyme‐associated variants are associated with liver disease risk (Figure 4), likely due to either a lack of power or the biological difference between liver enzyme variation and liver disease. For example, variant rs112574791 from gene glutamic pyruvic transaminase (GPT), which encodes cytosolic ALT, is strongly associated with lower serum ALT levels yet not liver disease (*p*_ALT_ = 1.27 × 10^−105^, *p*_any_liver_disease_ > 0.1). Interestingly, variants with significant BMI interactions ranked higher among liver disease associations compared with ALT‐associated variants without BMI interactions (*p*_INT_ ≥ 5 × 10^−8^, Wilcoxon's rank test, *p* = 6.78 × 10^−7^, Table S10).

Figure 4Scatter plots of ALT (AST) and liver disease association signals. (a) Scatter plots of ALT and liver disease associations with COJO selected independent variants only (*N* = 300). (b) Scatter plots of AST and liver disease associations with COJO selected independent variants only (*N* = 336). (c) Scatter plots of ALT and liver disease associations. (d) Scatter plots of AST and liver disease associations.  Genome‐wide significant BMI interaction variants are highlighted in red. ALT, alanine aminotransferase; AST, aspartate aminotransferase

**Figure:**
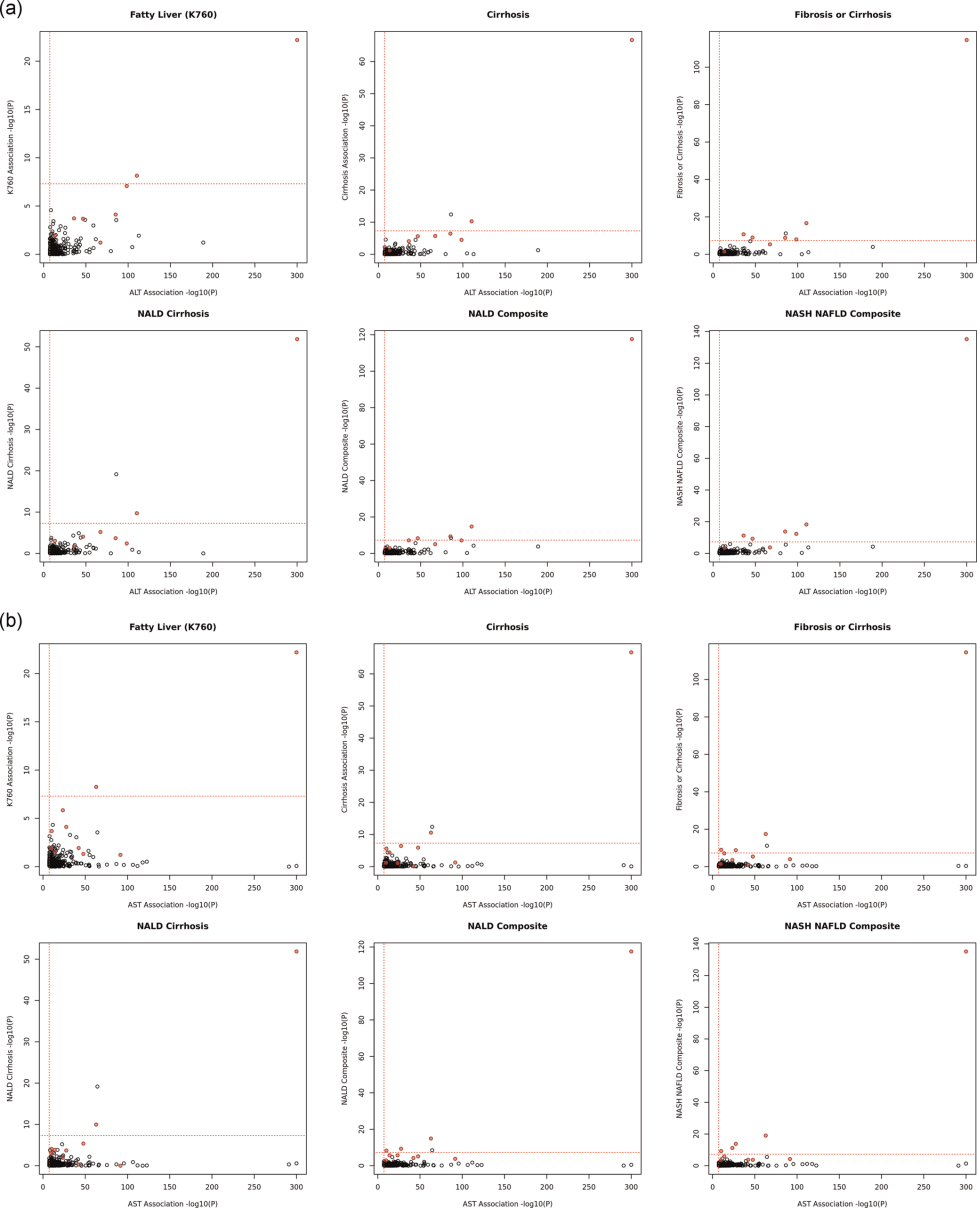

**Figure:**
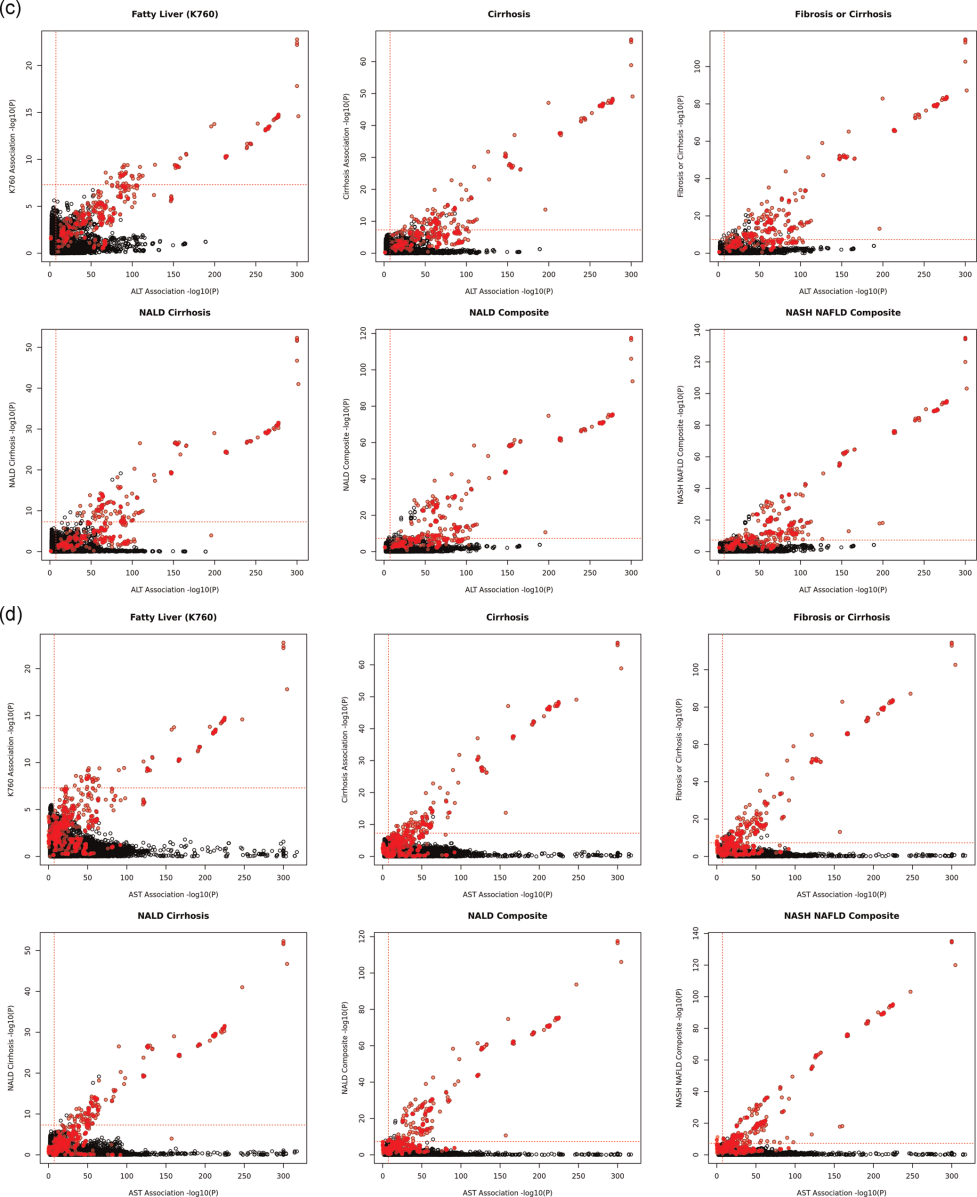

### 3.7 ALT, AST PRS, and liver disease

PRS were constructed using independent liver enzyme‐associated variants at different BMI‐interaction significance thresholds. A scheme of the constructed PRS is shown in Figure S8. PRS from ALT‐associated variants with significant BMI interactions (PRS9, 9 variants with *p*_ALT_ < 5 × 10^−8^, *p*_BMI_INT_ < 5 × 10^−8^) are strongly associated with liver disease, for example, NASH‐NALD composite (OR = 1.39, *p* = 3.91 × 10^−33^). PRS from ALT‐associated variants without significant BMI interactions (PRS87, 87 variants *p*_ALT_ < 5 × 10^−8^, *p*_BMI_INT_ > 0.5) had weaker effects and were less significantly associated (OR = 1.13, *p* = 8.10 × 10^−6^). This pattern of association with ALT polygenic scores was consistent for other liver disease traits and for polygenic scores built using AST association signals (Table S11).

## 4 DISCUSSION

Serum ALT and AST are commonly measured biomarkers of clinical importance. Serum ALT and AST levels have been analyzed together in genome‐wide association studies to shed light on the genetic etiology of liver damage and pathogenesis. ALT is primarily expressed in the liver and elevated serum ALT level is usually an indicator of liver damage or disease. AST is expressed in the liver, but it is also expressed in other organs including heart and skeletal muscle. Therefore, AST level elevation is not specifically indicative of liver damage or disease.

In this study, 11 million genetic markers were analyzed with serum ALT and AST levels in 388k European individuals. It is the largest GWAS of liver enzymes to date. After conditional analysis (GCTA COJO), 300 serum ALT and 336 AST independent significant associations were identified, including previously reported associations, for example, *PNPLA3*, *HSD17B13,* and *MARC1*. In addition, 81 serum ALT and 61 AST novel associations are identified, offering an important step forward in understanding the genetic architecture of serum ALT and AST levels.

The most significant ALT novel signal in this study was an intronic variant in *PPARG* (rs13083375, Figure S3 and Table S3), which is in strong LD (*R* ^2^ = 0.98) with a coding variant in exon 2 of the gene (rs1801282, p.P12A). In our analysis, rs13083375 and its proxy coding variant are associated with lower ALT levels. *PPARG* encodes a transcription factor that regulates adipocyte differentiation, adipogenesis, and lipid metabolism (Altshuler et al., 2000; O'Leary et al., 2016). In addition, *PPARG* is also expressed in liver hepatocytes. Transcriptional activation of PPARG in the liver has been shown to induce adipogenic mechanisms to store fatty acids in liver lipid droplets and therefore may be linked to the progression of NAFLD (Lee et al., 2018). Previous studies suggested that liver‐specific deletion of *PPARG* in mouse hepatocytes protects against development of steatosis (Matsusue et al., 2003). In our analysis, the variant did not exhibit a significant protective effect against NAFLD (OR = 0.96, *p* = 9.87 × 10^−2^) likely due to a lack of power.

Of the novel ALT‐ and AST‐associated variants, a missense variant in the gene *MTTP* has the most significant association with liver disease traits albeit suggestively significant (rs3816873, p.I128T, OR = 0.921, *p* = 3.33 × 10^−5^). Microsomal triglyceride transfer protein *(MTTP)* encodes a triglyceride transfer protein expressed in liver and has been implicated in lipoprotein assembly and lipid removal from hepatocytes (O'Leary et al., 2016). In UKB, rs3816873 is also modestly associated with lower LDL (*p* = 4.80 × 10^−6^) and APOB (*p* = 1.20 × 10^−6^) (data not shown). Other studies have shown that inhibition of MTTP may lead to hepatic steatosis (Bernard et al., 2000; Hashemi et al., 2011; Namikawa et al., 2004; Pereira et al., 2011). Collectively these results suggest that rs3816873 potentially modifies MTTP function and support its modulation to modify liver disease risk.

Among novel associations, we found multiple variants mapping to genes involved in lipid and adiposity metabolism, for example, haptoglobin‐related protein (*HPR*), serine palmitoyltransferase long chain base subunit 3 (*SPTLC3*), and ATP binding cassette subfamily G member 5 (*ABCG5*) (Tables S3 and S4). Although these observations support the role of lipid and adiposity metabolism contributing to liver damage (Fabbrini et al., 2010; Parekh & Anania, 2007), additional studies are needed to provide stronger genetic evidence that significantly supports a role for these genes in liver disease pathogenesis.

To better understand the impact of obesity on the genetic risk for liver damage and disease, we performed a GWIS exploring the modifying effects of BMI on serum ALT and AST genetic associations. The most significant signal was a missense variant (rs738409, p.I148M) in the gene *PNPLA3*. Relative to individuals in the lower BMI quartile (bottom 25%, BMI < 24.13 kg/m^2^), the per allele effect for variant rs738409 was more than ten times greater among individuals within the higher BMI quartile (top 25%, BMI > 29.82 kg/m^2^) (Figure 2). This observation is consistent with previous genetic analyses of *PNPLA3* (Giudice et al., 2011; Mann & Anstee, 2017; Stojkovic et al., 2014) and supports the synergistic effect between *PNPLA3* p.I148M and obesity. In addition, significant BMI modifying associations were also observed in multiple genes that have been evaluated as therapeutic targets for NAFLD. For example, ALT‐associated variants in *HSD17B13* and *MARC1* have stronger allelic effects in the higher BMI quartile (Figure 2). In contrast to these associations, the novel BMI‐ALT interaction association near gene cytochrome P450 family 7 subfamily A member 1 (*CYP7A1*) was observed only in individuals with a higher BMI. No effect is observed in low BMI individuals. Previous GWAS identified strong associations between *CYP7A1* and apolipoprotein B, triglyceride, and cholesterol levels (Richardson et al., 2020; Ripatti et al., 2020). This is the first genetic evidence of a BMI‐dependent ALT association. Although the mechanism of action that explains this association pattern is not clear, *CYP7A1* encodes a protein that catalyzes the first reaction in the cholesterol catabolic pathway and converts cholesterol to bile acids, which is the primary mechanism for the removal of cholesterol from the body (O'Leary et al., 2016). Taken together, these BMI‐dependent signals highlight how interaction analyses can improve our understanding of genetic effects on phenotypes by testing across different degrees of exposure and also show how we can improve our knowledge about the therapeutic potential of targets like *HSD17B13* and *MARC1* under these different conditions.

Our analysis also demonstrates how interaction analyses can inform our understanding about the therapeutic potential of novel association targets under certain genetic background. For example, we tested independent ALT and AST signals in a genetic interaction model with the *PNPLA3* coding variant p.I148M, a well‐established common variant (MAF_EUR_ = 21%) that confers strong susceptibility to NAFLD (Lin et al., 2014). In our targeted interaction screen, we found variants from *HSD17B13* significantly reduce the *PNPLA3* p.I148M allelic effect on ALT by 21%. In addition, this interaction has a greater effect within the higher BMI quartile (Figure 3). Despite the exact biological mechanism of the PNPLA3‐HSD17B13 interaction is not clear, these results suggest that targeting *HSD17B13* may reduce the risk of liver disease in those with a higher risk conferred by *PNPLA3* p.I148M, and that the *HSD17B13* protective potential may be stronger in individuals with a high BMI. On the other hand, variants in *MARC1* and other signals did not significantly interact with *PNPLA3* variant and therefore the mechanism could be independent from *PNPLA3* p.I148M.

In our tissue expression analysis, genes mapped to ALT‐associated variants were significantly upregulated in multiple tissues including liver, adipose tissue, and lung (Figure S6). Genes mapped to AST‐associated variants were found to be widely expressed across adipose tissue, lung, nerve, and liver (Figure S7). Notably, genes mapped to ALT‐ and AST‐associated variants with significant BMI interactions are significantly upregulated in liver and adipose tissue only. Although it is unclear how adiposity expression enriched genes could influence the pathogenesis of liver disease, it has been hypothesized that free fatty acids and adipokines released from adipose tissue increases the liver exposure to fat accumulation and therefore contribute to fatty liver disease risk (Jakobsen et al., 2007). These observations are consistent overall with the biological functions of ALT and AST, and also suggest that genes, with effects modified by BMI, may be important in this biological process by increasing the risk for liver damage and disease.

Significant ALT and AST variants have varying association significance with liver disease traits (Figure 4 and Tables S5 and S6). Interestingly, most of the significant BMI interaction signals were at least suggestively associated (*p* < 1.48 × 10^−4^, Bonferroni's correction) with NAFLD with consistent effect directions. In addition, Wilcoxon's rank test suggested that significant BMI interaction variants have stronger significance in liver disease associations (Table S10) relative to variants not significantly modified by BMI. Furthermore, polygenic analysis of serum ALT‐associated variants with significant BMI interactions are strongly associated with liver disease, yet ALT variants without BMI interaction effects have a weaker and less significant effect on liver disease risk. For example, among 300 independent ALT significant signals, 8 signals are genome‐wide significant with BMI interactions (*p*_INT_ < 5 × 10^−8^), and 87 signals are absent of BMI interactions (*p*_INT_ > 0.5). PRS based on the 8 signals are strongly associated with nonalcoholic liver disease (*p* = 2.54 × 10^−23^, OR = 1.40), yet the PRS based on 87 ALT signals have much weaker associations (*p* = 1.38 × 10^−4^, OR = 1.14). Several ALT and AST PRSs based on varying BMI interaction *p* values were tested and suggested a similar trend (Table S11). While larger samples sizes are required to determine if any of the individual variants identified in our analysis are significant risk factors for liver disease risk, collectively, the burden of serum ALT and AST variants modified by BMI are more likely to associate with liver disease traits. In other words, interaction models may help prioritize genes targeting liver diseases such as NAFLD.

While this study focused on individuals of European ancestry, BMI and fatty liver disease risk vary across ancestry groups (Ogden et al., 2014; Setiawan et al., 2016). GWAS and GWIS analyses in other ancestral populations will be necessary to comprehensively understand the global contribution of genetic factors to fatty liver disease risk. Including more diverse populations with variable distributions of BMI and incidences of fatty liver disease will enhance the discovery of genetic risk factors and advance our understanding of how BMI modifies the risk of liver disease specifically in these populations.

In summary, this study presents the largest genome‐wide association analysis of ALT and AST to date, and the first genome‐wide interaction screening of BMI interactions with these traits. The identified novel associations represent a substantial advance in understanding of the genetic architecture of serum ALT and AST levels, which may help explain the biological mechanism of liver disease and damage. The identification of multiple significant BMI interaction signals solidifies the role of adiposity in liver disease. Furthermore, we observed that ALT and AST associations with significant BMI interactions are also more likely to associate with liver disease traits. Taken together, the results may contribute to novel therapeutic target identification, and also shed light on precision medicine strategy for liver disease patient care.

## LIST OF AUTHOR NAMES AND CONTRIBUTIONS

**Geisinger Banner and Contribution Statements**

All authors/contributors are listed in alphabetical order.

Lance J. Adams^1^, Jackie Blank^1^, Dale Bodian^1^, Derek Boris^1^, Adam Buchanan^1^, David J. Carey^1^, Ryan D. Colonie^1^, F. Daniel Davis^1^, Dustin N. Hartzel^1^, Melissa Kelly^1^, H. Lester Kirchner^1^, Joseph B. Leader^1^, David H. Ledbetter^1^, Ph.D., J. Neil Manus^1^, Christa L. Martin^1^, Michelle Meyer^1^, Tooraj Mirshahi^1^, Matthew Oetjens^1^, Thomas Nate Person^1^, Christopher Still^1^, Natasha Strande^1^, Amy Sturm^1^, Jen Wagner^1^, Marc Williams^1^

Contribution: Development and validation of clinical phenotypes used to identify study participants and (when applicable) controls.

Affiliation: 1. Geisinger, Danville, PA.

**Regeneron Genetics Center Banner Author List and Contribution Statements**

All authors/contributors are listed in alphabetical order.

**RGC Management and Leadership Team**

Goncalo Abecasis, Ph.D., Aris Baras, M.D., Michael Cantor, M.D., Giovanni Coppola, M.D., Aris Economides, Ph.D., Luca A. Lotta, M.D., Ph.D., John D. Overton, Ph.D., Jeffrey G. Reid, Ph.D., Alan Shuldiner, M.D.

Contribution: All authors contributed to securing funding, study design and oversight. All authors reviewed the final version of the manuscript.

**Sequencing and Lab Operations**

Christina Beechert, Caitlin Forsythe, M.S., Erin D. Fuller, Zhenhua Gu, M.S., Michael Lattari, Alexander Lopez, M.S., John D. Overton, Ph.D., Thomas D. Schleicher, M.S., Maria Sotiropoulos Padilla, M.S., Louis Widom, Sarah E. Wolf, M.S., Manasi Pradhan, M.S., Kia Manoochehri, Ricardo H. Ulloa

Contribution: C.B., C.F., A.L., and J.D.O. performed and are responsible for sample genotyping. C.B, C.F., E.D.F., M.L., M.S.P., L.W., S.E.W., A.L., and J.D.O. performed and are responsible for exome sequencing. T.D.S., Z.G., A.L., and J.D.O. conceived and are responsible for laboratory automation. M.P., K.M., R.U., and J.D.O are responsible for sample tracking and the library information management system.

**Clinical Informatics**

Nilanjana Banerjee, Ph.D., Michael Cantor, M.D. M.A., Ashish Yadav, Deepika Sharma, MHI.

Contribution: All authors contributed to the development and validation of clinical phenotypes used to identify study subjects and (when applicable) controls.

**Genome Informatics**

Xiaodong Bai, Ph.D., Suganthi Balasubramanian, Ph.D., Andrew Blumenfeld, Boris Boutkov, Ph.D., Gisu Eom, Lukas Habegger, Ph.D., Alicia Hawes, B.S., Shareef Khalid, Olga Krasheninina, M.S., Rouel Lanche, Adam J. Mansfield, B.A., Evan K. Maxwell, Ph.D., Mrunali Nafde, Sean O'Keeffe, M.S., Max Orelus, Razvan Panea, Ph.D., Tommy Polanco, B.A., Ayesha Rasool, M.S., Jeffrey G. Reid, Ph.D., William Salerno, Ph.D., Jeffrey C. Staples, Ph.D.

Contribution: X.B., A.H., O.K., A.M., S.O., R.P., T.P., A.R., W.S. and J.G.R. performed and are responsible for the compute logistics, analysis and infrastructure needed to produce exome and genotype data. G.E., M.O., M.N. and J.G.R. provided compute infrastructure development and operational support. S.B., S.K., and J.G.R. provide variant and gene annotations and their functional interpretation of variants. E.M., J.S., R.L., B.B., A.B., L.H., J.G.R. conceived and are responsible for creating, developing, and deploying analysis platforms and computational methods for analyzing genomic data.

**Research Program Management**

Marcus B. Jones, Ph.D., Michelle G. LeBlanc, Ph.D., Jason Mighty, Ph.D., Lyndon J. Mitnaul, Ph.D.

Contribution: All authors contributed to the management and coordination of all research activities, planning and execution. All authors contributed to the review process for the final version of the manuscript.

## CONFLICT OF INTERESTS

Chuan Gao, Anthony Marcketta, Joshua D. Backman, Colm O'Dushlaine, Jeffrey Staples, Manuel Allen Revez Ferreira, Luca A. Lotta, John D. Overton, Jeffrey G. Reid, Aris Baras, Gonçalo Abecasis, Alan R. Shuldiner, Cristopher V. Van Hout, Shane McCarthy are current or former employees and/or stockholders of Regeneron Genetics Center. Chuan Gao is a current employee of Icahn School of Medicine at Mount Sinai, but work was conducted at Regeneron Genetics Center. Summary statistics of all independent significant signals are available in Supporting Information Tables.

## Supporting information

Supporting information.

Supporting information.

## Data Availability

Summary statistics of all independent significant signals are available in Supporting Information Tables.

## References

[gepi22392-bib-0001] Abul‐Husn, N. S., Cheng, X., Li, A. H., Xin, Y., Schurmann, C., Stevis, P., Liu, Y., Kozlitina, J., Stender, S., Wood, G. C., Stepanchick, A. N., Still, M. D., McCarthy, S., O'Dushlaine, C., Packer, J. S., Balasubramanian, S., Gosalia, N., Esopi, D., Kim, S. Y., … Dewey, F. E. (2018). A protein‐truncating HSD17B13 variant and protection from chronic liver disease. New England Journal of Medicine, 378(12), 1096–1106. 10.1056/NEJMoa1712191 PMC6668033 29562163

[gepi22392-bib-0002] Adams, L. A., Lymp, J. F., St, Sauver, J., Sanderson, S. O., Lindor, K. D., Feldstein, A., & Angulo, P. (2005). The natural history of nonalcoholic fatty liver disease: A population‐based cohort study. Gastroenterology, 129(1), 113–121. 10.1053/j.gastro.2005.04.014 16012941

[gepi22392-bib-0003] Altshuler, D., Hirschhorn, J. N., Klannemark, M., Lindgren, C. M., Vohl, M. C., Nemesh, J., Lane, C. R., Schaffner, S. F., Bolk, S., Brewer, C., Tuomi, T., Gaudet, D., Hudson, T. J., Daly, M., Groop, L., & Lander, E. S. (2000). The common PPARgamma Pro12Ala polymorphism is associated with decreased risk of type 2 diabetes. Nature Genetics, 26(1), 76–80. 10.1038/79216 10973253

[gepi22392-bib-0004] Bernard, S., Touzet, S., Personne, I., Lapras, V., Bondon, P. J., Berthezene, F., & Moulin, P. (2000). Association between microsomal triglyceride transfer protein gene polymorphism and the biological features of liver steatosis in patients with type II diabetes. Diabetologia, 43(8), 995–999. 10.1007/s001250051481 10990076

[gepi22392-bib-0005] Bulik‐Sullivan, B. K., Loh, P. R., Finucane, H. K., Ripke, S., Yang, J., Schizophrenia Working Group of the Psychiatric Genomics, C., Schizophrenia Working Group of the Psychiatric Genomics, C., Patterson, N., Daly, M. J., Price, A. L., & Neale, B. M. (2015). LD Score regression distinguishes confounding from polygenicity in genome‐wide association studies. Nature Genetics, 47(3), 291–295. 10.1038/ng.3211 25642630 PMC4495769

[gepi22392-bib-0006] Buniello, A., MacArthur, J., Cerezo, M., Harris, L. W., Hayhurst, J., Malangone, C., Mcmahon, A., Morales, J., Mountjoy, E., Sollis, E., Suveges, D., Vrousgou, O., Whetzel, P. L., Amode, R., Guillen, J. A., Riat, H. S., Trevanion, S. J., Hall, P., Junkins, H., … Parkinson, H. (2019). The NHGRI‐EBI GWAS Catalog of published genome‐wide association studies, targeted arrays and summary statistics 2019. Nucleic Acids Research, 47(D1), D1005–D1012. 10.1093/nar/gky1120 30445434 PMC6323933

[gepi22392-bib-0007] Bycroft, C., Freeman, C., Petkova, D., Band, G., Elliott, L. T., Sharp, K., Motyer, A., Vukcevic, D., Delaneau, O., O'Connell, J., Cortes, A., Welsh, S., Young, A., Effingham, M., McVean, G., Leslie, S., Allen, N., Donnelly, P., & Marchini, J. (2018). The UK Biobank resource with deep phenotyping and genomic data. Nature, 562(7726), 203–209. 10.1038/s41586-018-0579-z 30305743 PMC6786975

[gepi22392-bib-0008] Das, S., Forer, L., Schönherr, S., Sidore, C., Locke, A. E., Kwong, A., Vrieze, S. I., Chew, E. Y., Levy, S., McGue, M., Schlessinger, D., Stambolian, D., Loh, P. R., Iacono, W. G., Swaroop, A., Scott, L. J., Cucca, F., Kronenberg, F., Boehnke, M., … Fuchsberger, C. (2016). Next‐generation genotype imputation service and methods. Nature Genetics, 48(10), 1284–1287. 10.1038/ng.3656 27571263 PMC5157836

[gepi22392-bib-0009] Dewey, F. E., Murray, M. F., Overton, J. D., Habegger, L., Leader, J. B., Fetterolf, S. N., O'dushlaine, C., Van Hout, C. V., Staples, J., Gonzaga‐Jauregui, C., Metpally, R., Pendergrass, S. A., Giovanni, M. A., Kirchner, H. L., Balasubramanian, S., Abul‐Husn, N. S., Hartzel, D. N., Lavage, D. R., Kost, K. A., … Carey, D. J. (2016). Distribution and clinical impact of functional variants in 50,726 whole‐exome sequences from the DiscovEHR study. Science, 354(6319), 10.1126/science.aaf6814 28008009

[gepi22392-bib-0010] Emdin, C. A., Haas, M. E., Khera, A. V., Aragam, K., Chaffin, M., Klarin, D., Hindy, G., Jiang, L., Wei, W. Q., Feng, Q., Karjalainen, J., Havulinna, A., Kiiskinen, T., Bick, A., Ardissino, D., Wilson, J. G., Schunkert, H., McPherson, R., Watkins, H., … Kathiresan, S. (2020). A missense variant in Mitochondrial Amidoxime Reducing Component 1 gene and protection against liver disease. PLOS Genetics, 16(4), e1008629. 10.1371/journal.pgen.1008629 32282858 PMC7200007

[gepi22392-bib-0011] Fabbrini, E., Sullivan, S., & Klein, S. (2010). Obesity and nonalcoholic fatty liver disease: Biochemical, metabolic, and clinical implications. Hepatology, 51(2), 679–689. 10.1002/hep.23280 20041406 PMC3575093

[gepi22392-bib-0012] Giudice, E. M., Grandone, A., Cirillo, G., Santoro, N., Amato, A., Brienza, C., Savarese, P., Marzuillo, P., & Perrone, L. (2011). The association of PNPLA3 variants with liver enzymes in childhood obesity is driven by the interaction with abdominal fat. PLOS One, 6(11), e27933. 10.1371/journal.pone.0027933 22140488 PMC3225386

[gepi22392-bib-0013] GTEx Consortium (2015). Human genomics. The Genotype‐Tissue Expression (GTEx) pilot analysis: Multitissue gene regulation in humans. Science, 348(6235), 648–660. 10.1126/science.1262110 25954001 PMC4547484

[gepi22392-bib-0014] Hashemi, M., Hoseini, H., Yaghmaei, P., Moazeni‐Roodi, A., Bahari, A., Hashemzehi, N., & Shafieipour, S. (2011). Association of polymorphisms in glutamate‐cysteine ligase catalytic subunit and microsomal triglyceride transfer protein genes with nonalcoholic fatty liver disease. DNA and Cell Biology, 30(8), 569–575. 10.1089/dna.2010.1162 21438662

[gepi22392-bib-0015] Jakobsen, M. U., Berentzen, T., Sorensen, T. I., & Overvad, K. (2007). Abdominal obesity and fatty liver. Epidemiologic Reviews, 29, 77–87. 10.1093/epirev/mxm002 17478441

[gepi22392-bib-0016] Kaplan, M. M. (2002). Alanine aminotransferase levels: What's normal? Annals of Internal Medicine, 137(1), 49–51. 10.7326/0003-4819-137-1-200207020-00012 12093245

[gepi22392-bib-0017] Lee, Y. K., Park, J. E., Lee, M., & Hardwick, J. P. (2018). Hepatic lipid homeostasis by peroxisome proliferator‐activated receptor gamma 2. Liver Research, 2(4), 209–215. 10.1016/j.livres.2018.12.001 31245168 PMC6594548

[gepi22392-bib-0018] Lin, Y. C., Chang, P. F., Chang, M. H., & Ni, Y. H. (2014). Genetic variants in GCKR and PNPLA3 confer susceptibility to nonalcoholic fatty liver disease in obese individuals. American Journal of Clinical Nutrition, 99(4), 869–874. 10.3945/ajcn.113.079749 24477042

[gepi22392-bib-0019] Makkonen, J., Pietilainen, K. H., Rissanen, A., Kaprio, J., & Yki‐Jarvinen, H. (2009). Genetic factors contribute to variation in serum alanine aminotransferase activity independent of obesity and alcohol: A study in monozygotic and dizygotic twins. Journal of Hepatology, 50(5), 1035–1042. 10.1016/j.jhep.2008.12.025 19303161

[gepi22392-bib-0020] Mann, J. P., & Anstee, Q. M. (2017). NAFLD: PNPLA3 and obesity: A synergistic relationship in NAFLD. Nature Reviews Gastroenterology & Hepatology, 14(9), 506–507. 10.1038/nrgastro.2017.74 28611479

[gepi22392-bib-0021] Matsusue, K., Haluzik, M., Lambert, G., Yim, S. H., Gavrilova, O., Ward, J. M., Brewer B, J.r, Reitman, M. L., & Gonzalez, F. J. (2003). Liver‐specific disruption of PPARgamma in leptin‐deficient mice improves fatty liver but aggravates diabetic phenotypes. Journal of Clinical Investigation, 111(5), 737–747. 10.1172/JCI172 PMC151902 12618528

[gepi22392-bib-0022] Moon, S., Kim, Y. J., Han, S., Hwang, M. Y., Shin, D. M., Park, M. Y., Lu, Y., Yoon, K., Jang, H. M., Kim, Y. K., Park, T. J., Song, D. S., Park, J. K., Lee, J. E., & Kim, B. J. (2019). The Korea Biobank Array: Design and identification of coding variants associated with blood biochemical traits. Scientific Reports, 9(1), 1382. 10.1038/s41598-018-37832-9 30718733 PMC6361960

[gepi22392-bib-0023] Namikawa, C., Shu‐Ping, Z., Vyselaar, J. R., Nozaki, Y., Nemoto, Y., Akisawa, N., Saibara, T., Hiroi, M., Enzan, H., & Onishi, S. (2004). Polymorphisms of microsomal triglyceride transfer protein gene and manganese superoxide dismutase gene in non‐alcoholic steatohepatitis. Journal of Hepatology, 40(5), 781–786. 10.1016/j.jhep.2004.01.028 15094225

[gepi22392-bib-0024] O'Leary, N. A., Wright, M. W., Brister, J. R., Ciufo, S., Haddad, D., McVeigh, R., Rajput, B., Robbertse, B., Smith‐White, B., Ako‐Adjei, D., Astashyn, A., Badretdin, A., Bao, Y., Blinkova, O., Brover, V., Chetvernin, V., Choi, J., Cox, E., Ermolaeva, O., … Pruitt, K. D. (2016). Reference sequence (RefSeq) database at NCBI: Current status, taxonomic expansion, and functional annotation. Nucleic Acids Research, 44(D1), D733–D745. 10.1093/nar/gkv1189 26553804 PMC4702849

[gepi22392-bib-0025] Ogden, C. L., Carroll, M. D., Kit, B. K., & Flegal, K. M. (2014). Prevalence of childhood and adult obesity in the United States, 2011‐2012. Journal of the American Medical Association, 311(8), 806–814. 10.1001/jama.2014.732 24570244 PMC4770258

[gepi22392-bib-0026] Parekh, S., & Anania, F. A. (2007). Abnormal lipid and glucose metabolism in obesity: implications for nonalcoholic fatty liver disease. Gastroenterology, 132(6), 2191–2207. 10.1053/j.gastro.2007.03.055 17498512

[gepi22392-bib-0027] Pereira, I. V., Stefano, J. T., & Oliveira, C. P. (2011). Microsomal triglyceride transfer protein and nonalcoholic fatty liver disease. Expert Review of Gastroenterology & Hepatology, 5(2), 245–251. 10.1586/egh.11.22 21476919

[gepi22392-bib-0028] Prins, B. P., Kuchenbaecker, K. B., Bao, Y., Smart, M., Zabaneh, D., Fatemifar, G., Luan, J., Wareham, N. J., Scott, R. A., Perry, J., Langenberg, C., Benzeval, M., Kumari, M., & Zeggini, E. (2017). Genome‐wide analysis of health‐related biomarkers in the UK Household Longitudinal Study reveals novel associations. Scientific Reports, 7(1), 11008. 10.1038/s41598-017-10812-1 28887542 PMC5591265

[gepi22392-bib-0029] Purcell, S., Neale, B., Todd‐Brown, K., Thomas, L., Ferreira, M. A., Bender, D., Maller, J., Sklar, P., de Bakker, P. I., Daly, M. J., & Sham, P. C. (2007). PLINK: A tool set for whole‐genome association and population‐based linkage analyses. American Journal of Human Genetics, 81(3), 559–575. 10.1086/519795 17701901 PMC1950838

[gepi22392-bib-0030] Rahmioglu, N., Andrew, T., Cherkas, L., Surdulescu, G., Swaminathan, R., Spector, T., & Ahmadi, K. R. (2009). Epidemiology and genetic epidemiology of the liver function test proteins. PLOS One, 4(2), e4435. 10.1371/journal.pone.0004435 19209234 PMC2636884

[gepi22392-bib-0031] Richardson, T. G., Sanderson, E., Palmer, T. M., Ala‐Korpela, M., Ference, B. A., Davey Smith, G., & Holmes, M. V. (2020). Evaluating the relationship between circulating lipoprotein lipids and apolipoproteins with risk of coronary heart disease: A multivariable Mendelian randomisation analysis. PLOS Medicine, 17(3), e1003062. 10.1371/journal.pmed.1003062 32203549 PMC7089422

[gepi22392-bib-0032] Ripatti, P., Rämö, J. T., Mars, N. J., Fu, Y., Lin, J., Söderlund, S., Benner, C., Surakka, I., Kiiskinen, T., Havulinna, A. S., Palta, P., Freimer, N. B., Widén, E., Salomaa, V., Tukiainen, T., Pirinen, M., Palotie, A., Taskinen, M. R., Ripatti, S., & FinnGen†. (2020). Polygenic Hyperlipidemias and Coronary Artery Disease Risk. Circulation: Genomic and Precision Medicine, 13(2), e002725. 10.1161/CIRCGEN.119.002725 32154731 PMC7176338

[gepi22392-bib-0033] Romeo, S., Kozlitina, J., Xing, C., Pertsemlidis, A., Cox, D., Pennacchio, L. A., Boerwinkle, E., Cohen, J. C., & Hobbs, H. H. (2008). Genetic variation in PNPLA3 confers susceptibility to nonalcoholic fatty liver disease. Nature Genetics, 40(12), 1461–1465. 10.1038/ng.257 18820647 PMC2597056

[gepi22392-bib-0034] Setiawan, V. W., Stram, D. O., Porcel, J., Lu, S. C., Le Marchand, L., & Noureddin, M. (2016). Prevalence of chronic liver disease and cirrhosis by underlying cause in understudied ethnic groups: The multiethnic cohort. Hepatology, 64(6), 1969–1977. 10.1002/hep.28677 27301913 PMC5115980

[gepi22392-bib-0035] Sharma, B., & John, S. (2019). Nonalcoholic steatohepatitis. StatPearls. 29262166

[gepi22392-bib-0036] Sinnott‐Armstrong, N., Tanigawa, Y., Amar, D., Mars, N., Daly, M. J., & Rivas, M. A. (2019). Genetics of 38 blood and urine biomarkers in the UK Biobank. BioRxiv, 53, 185–194. 10.1101/660506 PMC7867639 33462484

[gepi22392-bib-0037] Sookoian, S., & Pirola, C. J. (2015). Liver enzymes, metabolomics and genome‐wide association studies: from systems biology to the personalized medicine. World Journal of Gastroenterology, 21(3), 711–725. 10.3748/wjg.v21.i3.711 25624707 PMC4299326

[gepi22392-bib-0038] Spengler, E. K., & Loomba, R. (2015). Recommendations for diagnosis, referral for liver biopsy, and treatment of nonalcoholic fatty liver disease and nonalcoholic steatohepatitis. Mayo Clinic Proceedings, 90(9), 1233–1246. 10.1016/j.mayocp.2015.06.013 26219858 PMC4567478

[gepi22392-bib-0039] Staples, J., Qiao, D., Cho, M. H., Silverman, E. K., University of Washington Center for Mendelian, G., Nickerson, D. A., & Below, J. E. (2014). PRIMUS: Rapid reconstruction of pedigrees from genome‐wide estimates of identity by descent. American Journal of Human Genetics, 95(5), 553–564. 10.1016/j.ajhg.2014.10.005 25439724 PMC4225580

[gepi22392-bib-0040] Stojkovic, I. A., Ericson, U., Rukh, G., Riddestrale, M., Romeo, S., & Orho‐Melander, M. (2014). The PNPLA3 Ile148Met interacts with overweight and dietary intakes on fasting triglyceride levels. Genes and Nutrition, 9(2), 388. 10.1007/s12263-014-0388-4 24563329 PMC3968290

[gepi22392-bib-0041] Watanabe, K., Taskesen, E., van Bochoven, A., & Posthuma, D. (2017). Functional mapping and annotation of genetic associations with FUMA. Nature Communications, 8(1), 1826. 10.1038/s41467-017-01261-5 PMC5705698 29184056

[gepi22392-bib-0042] Willer, C. J., Li, Y., & Abecasis, G. R. (2010). METAL: Fast and efficient meta‐analysis of genomewide association scans. Bioinformatics, 26(17), 2190–2191. 10.1093/bioinformatics/btq340 20616382 PMC2922887

[gepi22392-bib-0043] Yki‐Jarvinen, H. (2014). Non‐alcoholic fatty liver disease as a cause and a consequence of metabolic syndrome. Lancet Diabetes and Endocrinology, 2(11), 901–910. 10.1016/S2213-8587(14)70032-4 24731669

[gepi22392-bib-0044] Young, K. A., Palmer, N. D., Fingerlin, T. E., Langefeld, C. D., Norris, J. M., Wang, N., Xiang, A. H., Guo, X., Williams, A. H., Chen, Y. I., Taylor, K. D., Rotter, J. I., Raffel, L. J., Goodarzi, M. O., Watanabe, R. M., & Wagenknecht, L. E. (2019). Genome‐wide association study identifies loci for liver enzyme concentrations in Mexican Americans: The GUARDIAN Consortium. Obesity (Silver Spring), 27(8), 1331–1337. 10.1002/oby.22527 31219225 PMC6656610

[gepi22392-bib-0045] Zhou, W., Nielsen, J. B., Fritsche, L. G., Dey, R., Gabrielsen, M. E., Wolford, B. N., Lefaive, J., VandeHaar, P., Gagliano, S. A., Gifford, A., Bastarache, L. A., Wei, W. Q., Denny, J. C., Lin, M., Hveem, K., Kang, H. M., Abecasis, G. R., Willer, C. J., & Lee, S. (2018). Efficiently controlling for case‐control imbalance and sample relatedness in large‐scale genetic association studies. Nature Genetics, 50(9), 1335–1341. 10.1038/s41588-018-0184-y 30104761 PMC6119127

