# Supplementary material for: Genome‐wide association analysis of serum alanine and aspartate aminotransferase, and the modifying effects of BMI in 388k European individuals
Source: Genet Epidemiol. 2021 Jun 29;45(6):664–81. doi: 10.1002/gepi.22392 (PMC8457092; doi:10.1002/gepi.22392)

SUPPLEMENTAL FIGURES

- Figure S1. Manhattan and QQ plots of ALT and AST associations.
- Figure S2. Regional LocusZoom plot of *PNPLA3* signal.
- Figure S3. Regional LocusZoom plot of *PPARG* signal.
- Figure S4. Regional LocusZoom plot of *MTTP* signal.
- Figure S5. Regional LocusZoom plot of *CYP7A1* signal.
- Figure S6. Tissue expression enrichment analysis of ALT signals.
- Figure S7. Tissue expression enrichment analysis of AST signals.
- Figure S8. Polygenic risk score construction scheme.

FIGURE S1. QQ PLOTS OF ALT AND AST ASSOCIATIONS.

Figure S1a. QQ plots of ALT main effect (left) and BMI-interaction (right) associations.

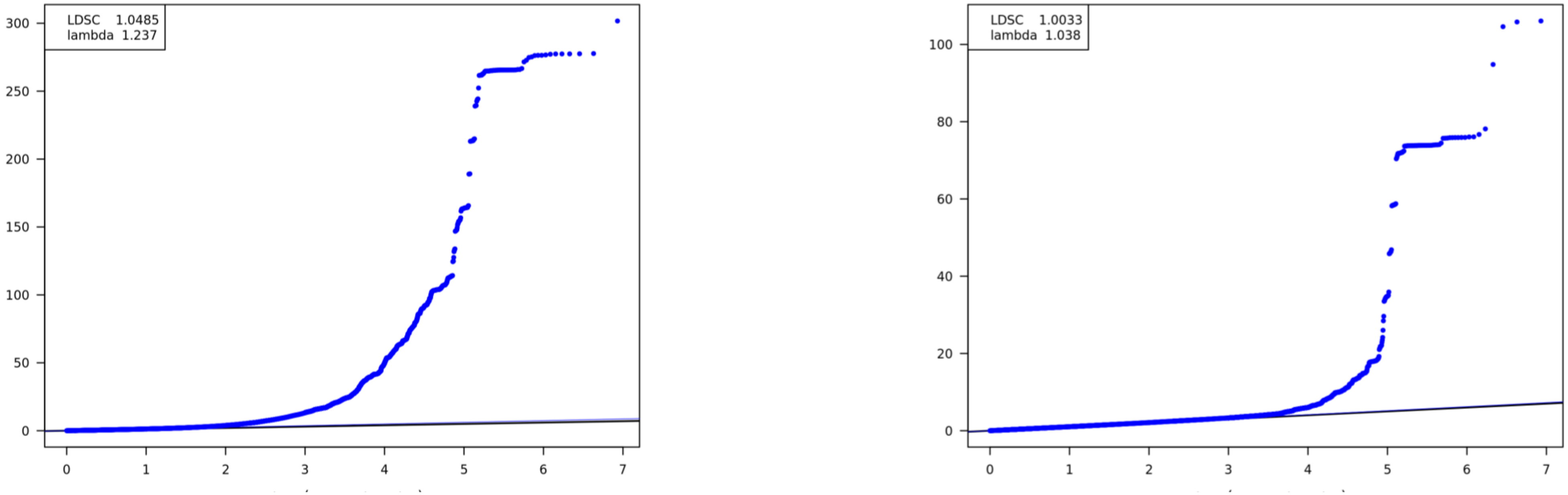

FIGURE S1. QQ PLOTS OF ALT AND AST ASSOCIATIONS.

Figure S1b. QQ plots of AST main effect (left) and BMI-interaction (right) associations.

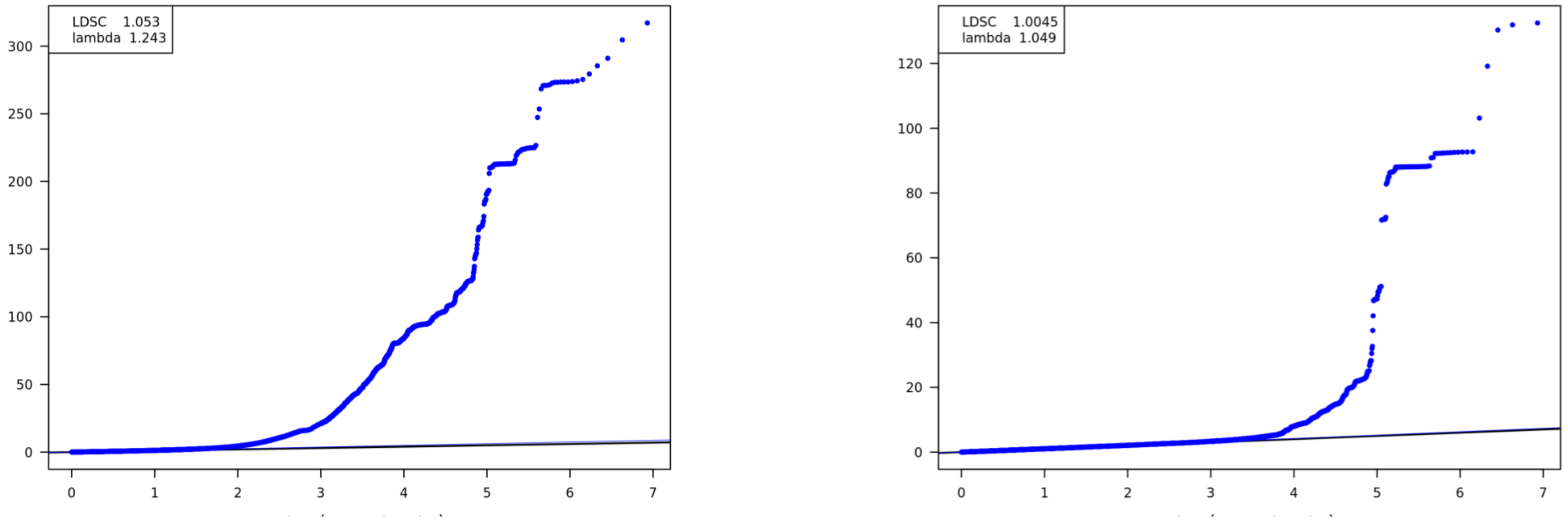

FIGURE S2. REGIONAL LOCUSZOOM PLOT OF *PNPLA3* SIGNAL.

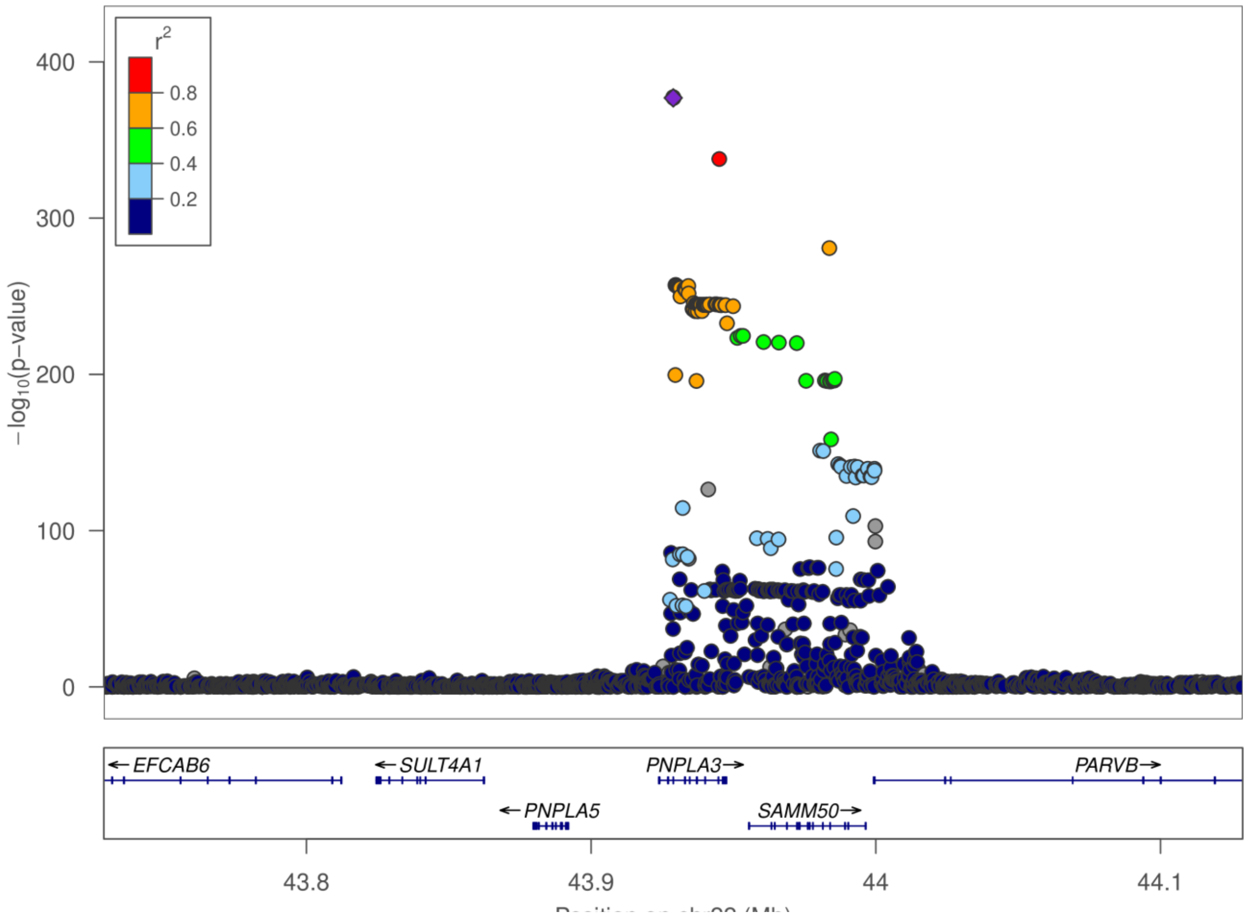

FIGURE S3. REGIONAL LOCUSZOOM PLOT OF *PPARG* SIGNAL.

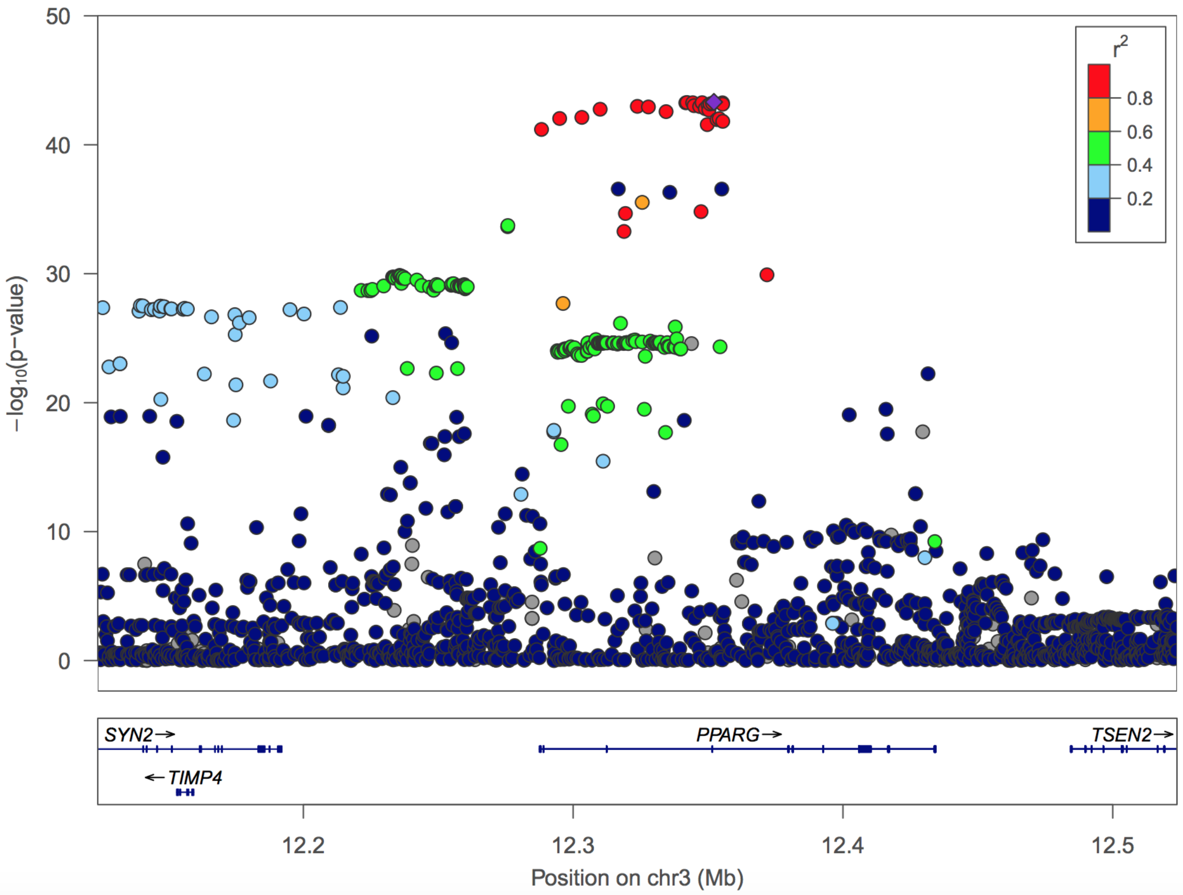

FIGURE S4. REGIONAL LOCUSZOOM PLOT OF *MTTP* SIGNAL.

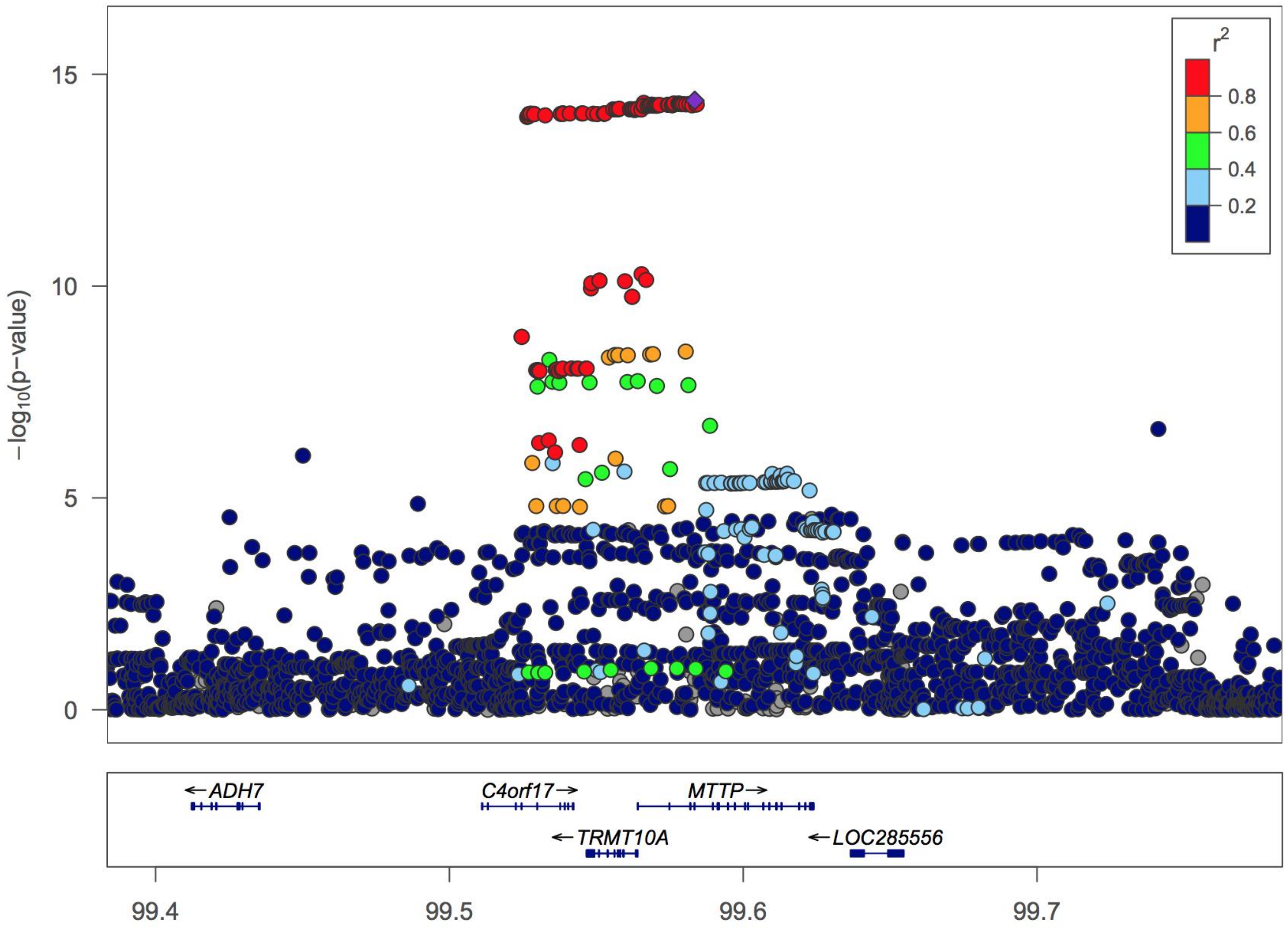

FIGURE S5. REGIONAL LOCUSZOOM PLOT OF *CYP7A1* SIGNAL.

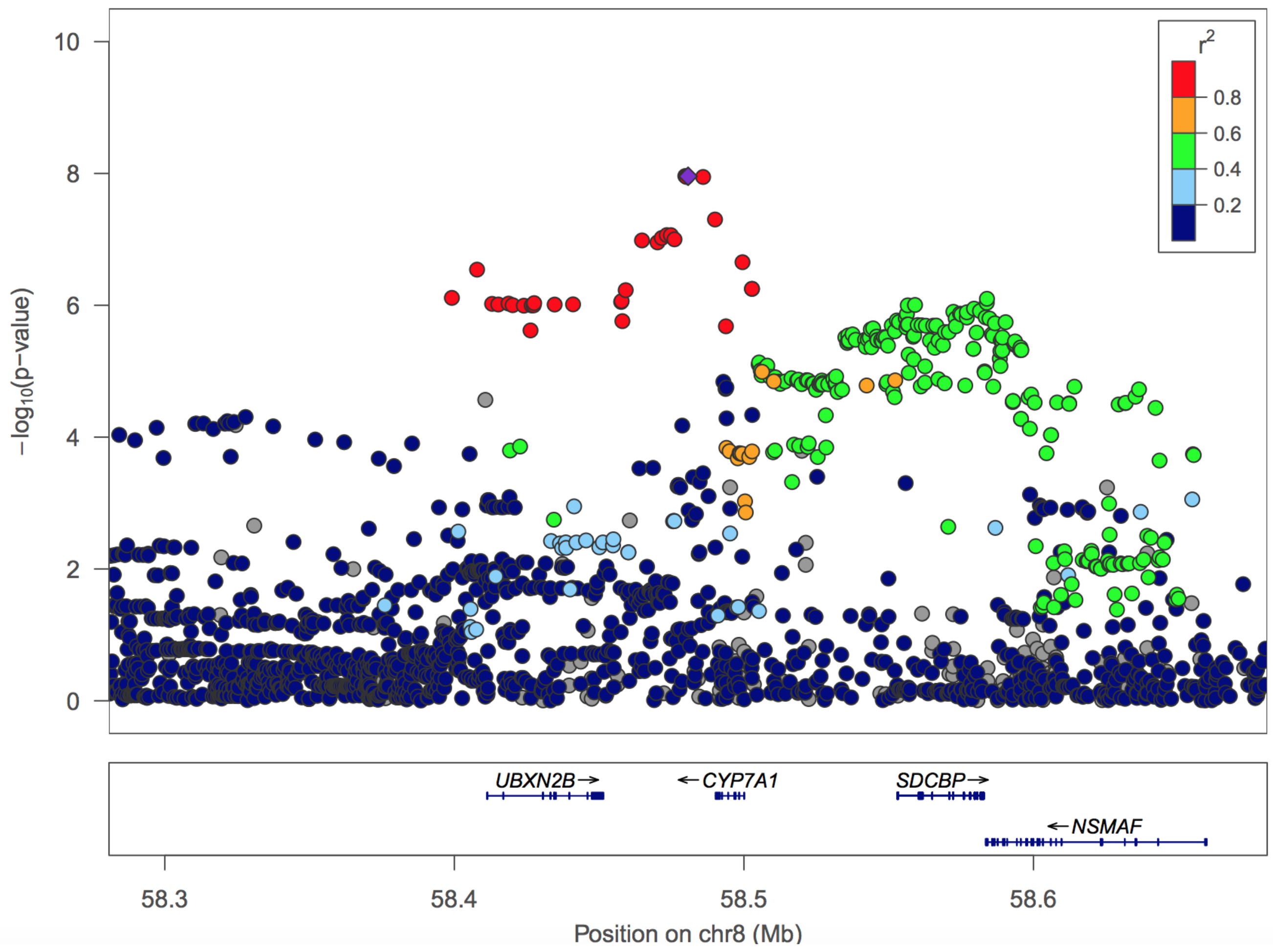

FIGURE S6. TISSUE EXPRESSION ENRICHMENT ANALYSIS OF ALT SIGNALS.

Figure 5a. Expression enrichment of ALT main effect signals

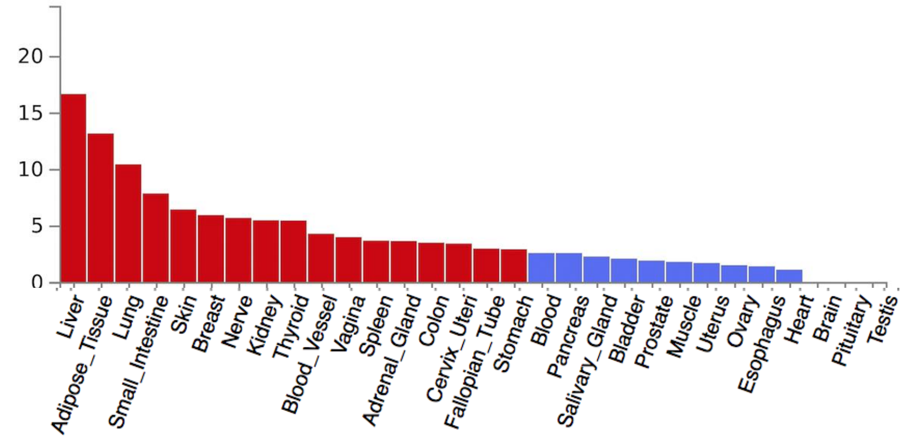

Figure 5b. Expression enrichment of previously reported ALT main effect signals

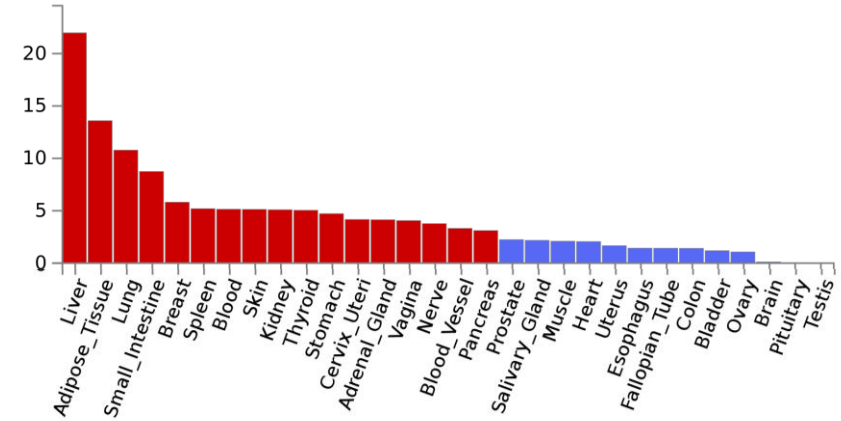

Figure 5c. Expression enrichment of novel ALT main effect signals

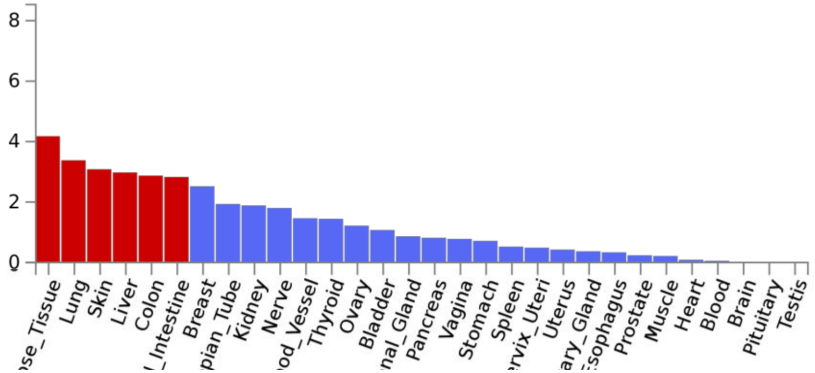

Figure 5d. Expression enrichment of ALT signals with BMI interactions

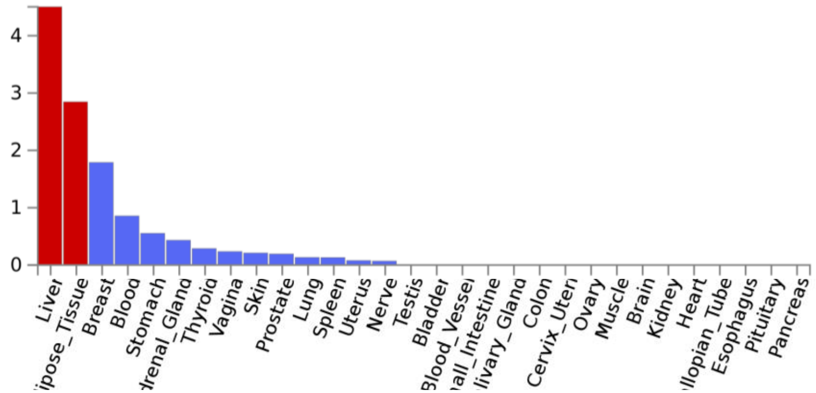

FIGURE S7. TISSUE EXPRESSION ENRICHMENT ANALYSIS OF AST SIGNALS.

Figure 6a. Expression enrichment of AST main effect signals

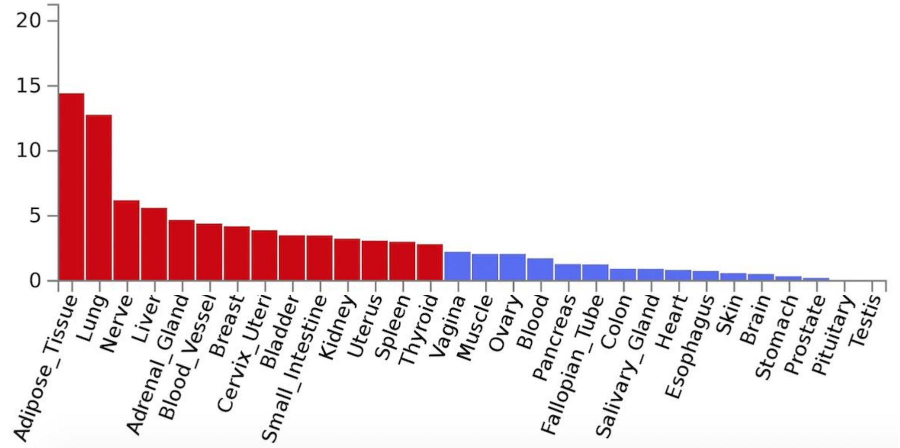

Figure 6b. Expression enrichment of previously reported AST main effect signals

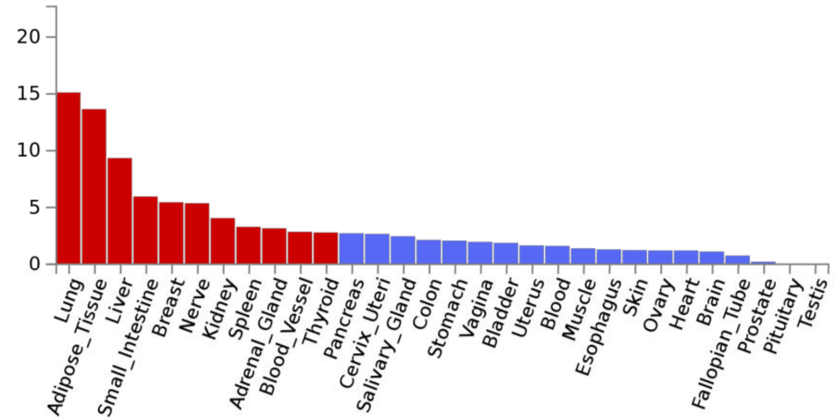

Figure 6c. Expression enrichment of novel AST main effect signals

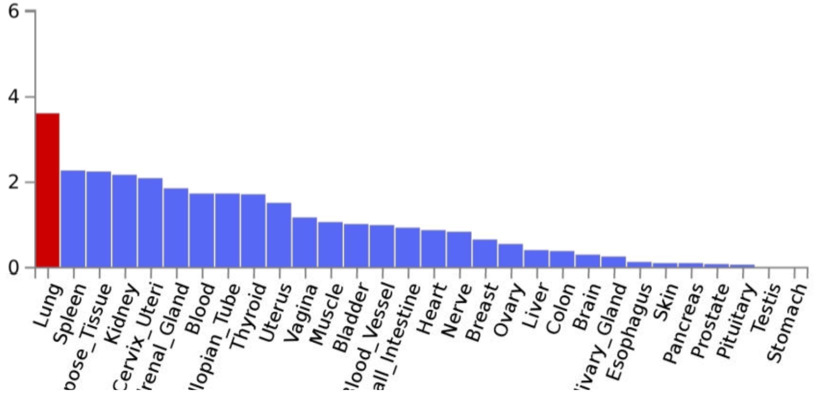

Figure 6d. Expression enrichment of AST signals with BMI interactions

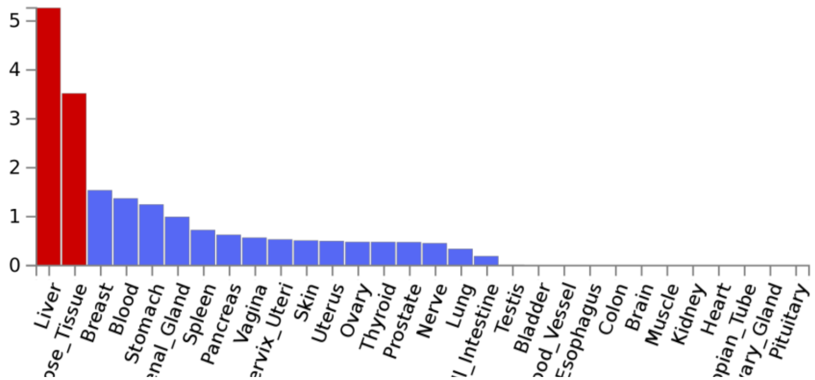

FIGURE S8. POLYGENIC RISK SCORE CONSTRUCTION SCHEME.

Figure S8a. ALT Polygenic risk score construction scheme.

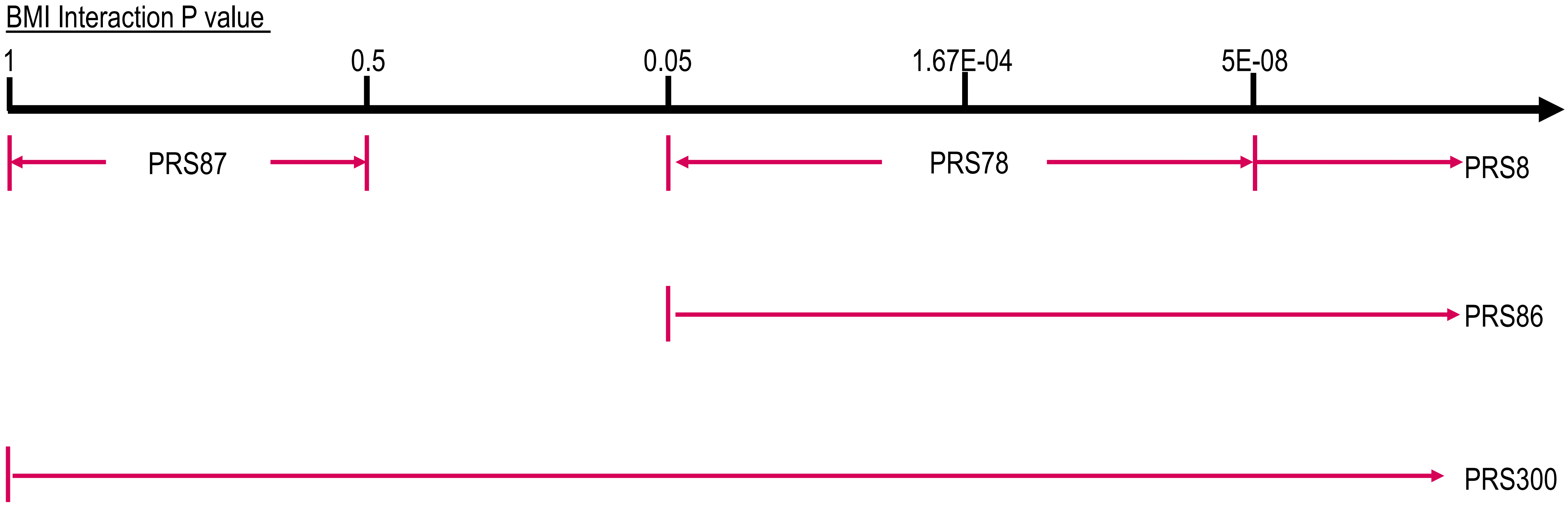

FIGURE S8. POLYGENIC RISK SCORE CONSTRUCTION SCHEME

Figure S8b. AST Polygenic risk score construction scheme.

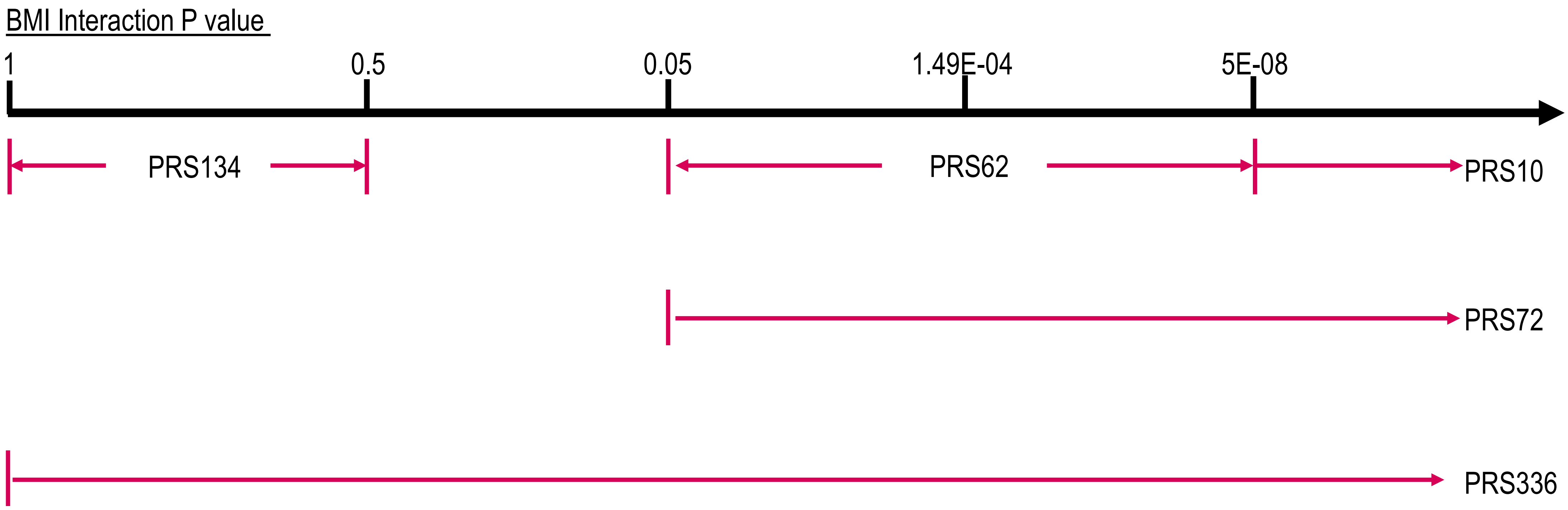

Supplement: Supplementary file 1 — Supporting information. [file GEPI-45-664-s001.pdf]
